# Nonequilibrium description of de novo biogenesis and transport through Golgi-like cisternae

**DOI:** 10.1038/srep38840

**Published:** 2016-12-19

**Authors:** Himani Sachdeva, Mustansir Barma, Madan Rao

**Affiliations:** 1 Department of Theoretical Physics, Tata Institute of Fundamental Research, Homi Bhabha Road, Mumbai, 400005, India; 2 Institute of Science and Technology, Am Campus 1, Klosterneuburg, A-3400, Austria; 3 TIFR Centre for Interdisciplinary Sciences, 21 Brundavan Colony, Narsingi, Hyderabad 500075, India; 4 Simons Centre for the Study of Living Machines, National Centre for Biological Sciences (TIFR), Bellary Road, Bangalore, 560065, India

## Abstract

A central issue in cell biology is the physico-chemical basis of organelle biogenesis in intracellular trafficking pathways, its most impressive manifestation being the biogenesis of Golgi cisternae. At a basic level, such morphologically and chemically distinct compartments should arise from an interplay between the molecular transport and chemical maturation. Here, we formulate analytically tractable, minimalist models, that incorporate this interplay between transport and chemical progression in physical space, and explore the conditions for *de novo* biogenesis of distinct cisternae. We propose new quantitative measures that can discriminate between the various models of transport in a *qualitative* manner–this includes measures of the dynamics in steady state and the dynamical response to perturbations of the kind amenable to live-cell imaging.

One of the striking features of eukaryotic cells, is the appearance of compartmentalization, especially under continual remodeling, such as in the endosomal or secretory trafficking pathways^1^,^2^. This naturally raises the question, are there robust self-organizational principles that lead to the emergence of chemically and morphologically distinct compartments in such dynamic situations^3^,^4^?

Such questions can be discussed in the context of the Golgi Apparatus, which consists of multiple stacks of chemically distinct, membrane-bound cisternae^5^. Starting from their point of synthesis in the endoplasmic reticulum (ER), lipids and proteins are transported vectorially through the polarized Golgi stacks towards the plasma membrane (PM), undergoing a sequence of enzymatic conversions during their progression. The interplay between vectorial transport and biochemical polarity appears quite generally across different cell types, though the number, shape and spatial extent of the Golgi cisternae may exhibit variation^6^.

A detailed knowledge of *individual molecular processes* notwithstanding, a step in understanding the *collective dynamics* of morphological and chemical identity is to construct minimalist theoretical models^7^,^8^,^9^,^10^,^11^,^12^ that take into account the essential microscopic dynamical and chemical processes in the Golgi, to arrive at general conditions for compartment biogenesis. Such an exercise would be useful in addressing the fundamental issue of whether the Golgi organelle is constructed from a pre-existing template or generated *de novo*, a result of self-organization^4^,^6^,^13^,^14^. In the process, it may lead us to revisit and make precise, the notion of an organelle.

Our minimalist theoretical framework incorporates the two classical competing models for intra-Golgi transport, (A) *Vesicular Transport (VT*) and (B) *Cisternal Progression and Maturation (CP*). In the VT model, cisternae are assumed to be stationary and temporally stable structures, each with a fixed set of enzymes. Molecules, packaged within vesicles, shuttle from one cisterna to the next, and get chemically modified by the resident enzymes. By contrast, in the CP model, there is a constant turnover of cisternae–new cisternae form by the fusion of incoming vesicles at the cis end, and move *as a whole* through the Golgi, carrying cargo molecules with them. Specific enzymes get attached to a cisterna in different stages of its progression, and modify its contents. The terminal (trans) cisterna is the site of extensive tubulation and budding, whereby processed biomolecules are released onto the PM^1^,^15^.

Experimental evidence in support of the two hypotheses, namely, VT and CP^16^,^17^,^18^ or their combinations^19^,^20^,^21^, is mixed at best. To our knowledge, except in the few cases, it has been difficult to implement an experimental strategy that can point unequivocally to a specific transport model. Indeed natural cellular realizations might have aspects of both these models. One of our objectives is to suggest new quantitative measures that can discriminate between various contending models. In order to do this, we construct a general multi-species model that encompasses essential features of both VT and CP and their many variants (such as the recently proposed ‘cisternal progenitor’ and ‘rim progression’ models^22^,^23^). Our generic framework allows us to take each of these models and explore its consequences in detail.

The rules governing the model are kept fairly general, with no assumptions of molecular constraints or selectivity, hoping to arrive at a good compromise between complexity and analytical tractability. The general model can be analyzed precisely in various limits, to determine the conditions under which one might obtain multiple compartments that are large, clearly separated, chemically distinct, statistically stable and robust to perturbations–properties that are desirable for organelle biogenesis. Further, we propose precise biophysical measurements, associated with subtle features of the dynamics in steady state and dynamical response to a variety of perturbations, that show *qualitatively* distinct signatures of the underlying transport mechanism.

## Model and Methods

Our general model is defined in terms of dynamical moves of “particles” (vesicles) each carrying a chemical species, denoted as *A, B, C*, …, on a 1-dimensional (1D) system represented here as a 1D lattice with *L* sites. This could describe the endosomal and secretory pathways where the primary transport, barring a few branching events, is along 1D. However for definiteness, we will henceforth use the terminology of the secretory pathway. Here, *L* is the length of the cytoplasmic region between the source/ER (site 0) and the sink/PM (site *L*). The position coordinate, which signifies the physical distance from the ER along the cis to trans axis of a single Golgi stack, is then a coordinate in real space and not a cisternal index (as assumed in ref. 24), and so exists even in the absence of cisternae. For convenience, we describe the dynamics on lattice points, but this is not crucial.

**Figure 1:**
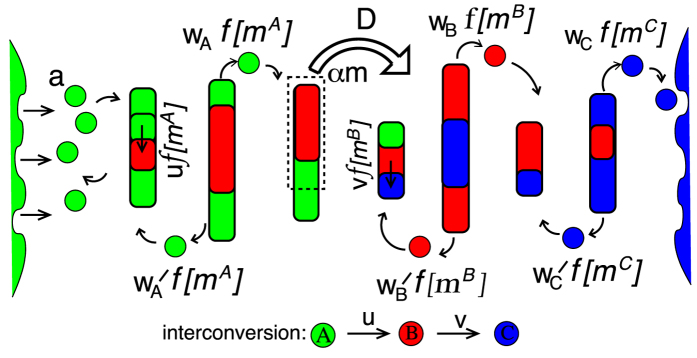
Schematic of the effective 1D transport model with three-species–*A, B* and *C* of particles (vesicles). The stochastic model includes injection from the ER (left) with rate *a*, fission-fusion (with rates 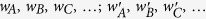) of vesicles, aggregate breakage (fraction *α*) and movement (rate *D*), chemical interconversion (rates *u, v*, …) and exit from the PM (right boundary) or recycling back to the ER (left boundary). The rates of transport, fission, interconversion depend on the amount or “mass” of the chemical species at the donor and acceptor sites through the flux-kernel *f*. Rates of various processes depicted in the figure are detailed in S1.1, Supplementary Information.

The model allows for (i) influx of unprocessed cargo at the left (ER) in the form of injection of A particles, (ii) outflux of processed molecules at the right (PM), i.e., exit of particles from the right boundary, (iii) recycling of particles back to the ER, i.e., exit of particles from the left, (iv) fusion of vesicles (particles) to form a cisterna (aggregate) and fusion to a preexisting cisternae, (v) fission of vesicles or large fragments (containing a fraction *α* of the mass) from cisternae, (vi) transport within the system either via single particle movement (vesicle exchange) and/or movement of cisternal fragments or of the entire aggregate of particles (cisternal movement), and finally, (vii) chemical transformation or processing of molecules in the aggregates, via the sequential interconversion *A* → *B* → *C…* of particles. For further details, see S1.1, Supplementary Information (also schematic in Fig. 1).

Bidirectional vesicular transport is modeled via asymmetric anterograde/retrograde particle movement rates, with *w*_*A*_ → *γ*_*A*_*w*_*A*_, 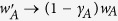, where *γ*_*A*_ parametrizes the asymmetry for A particles and so on (Fig. 1). The dependence of the asymmetry factors *γ*_*A*_, *γ*_*B*_, *γ*_*C*_ on the chemical species corresponds to differential degrees of recycling of *A, B, C*, … particles in the anterograde direction. The asymmetry factors could depend on the activity of GTPases such as CDC42^25^, and can be reliably estimated by comparing measured rates of retrograde and anterograde transport^26^. The two rates are found to be very similar for traffic between ER and cis-Golgi^26^, which is consistent with the significant vesicular recycling (25–40%) that we will need to assume in our model.

The rates of fission-fusion and chemical conversion depend on the amount (or “mass”) of chemical species in the donor (*m*_*i*_) and acceptor (*m*_*i*±1_) aggregates, through the flux-kernel, *f*[*m*_*i*_|*m*_*i*±1_]. There are many choices of the flux-kernel (see S2.2, SI for a discussion), but the form that we explore in detail is independent of the mass of the acceptor aggregate:


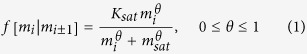


and corresponds to Michelis-Menten (MM) type of enzymatic kinetics for fission or chemical conversion, where we choose *θ* = 1/2. Thus, reaction rates increase as 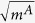, 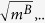 for small amounts of *A, B* in the parent aggregate, but become enzyme-limited and saturate to a constant value *K*_*sat*_ when the amount of *A, B* exceeds *m*_*sat*_.

Our choice of *θ* = 1/2 can be justified by first representing any 2D disc-like structure at position *i* along the cis to trans direction by its mass *m*_*i*_ and then assuming that only perimeter molecules participate in the flux. In principle, the saturation scale *m*_*sat*_ and even the exponent *θ* can be different for fission and conversion processes (see S2.2, SI for an example of structure formation in this case), but to keep the model analytically tractable we restrict them to be the same for both. This choice is not limiting and leads to structure formation and dynamics qualitatively similar to that in the more general model with varying *θ* and *m*_*sat*_ for the two processes. We have also studied flux-kernels which depend on the amount of the chemical species in the acceptor aggregate, and find that this also does does not qualitatively impact self-organisation, at least within a significant region of parameter space (see S2.4, SI).

In addition to vesicular transport, the model also allows for movement of larger structures at rate *D*, with *α* = 1 corresponding to cisternal progression and *α* < 1 to a cisternal-progenitor-like mechanism characterized by the scission and movement of cisternal fragments. When transport occurs solely via cisternal or sub-cisternal movement, then *αD* can be estimated as ~*L*/*τ*, where *τ* is the typical cargo transit time through a Golgi stack. For joint estimation of *D* with *w*_*A*_*K*_*sat*_ etc., which determine vesicular fluxes, more complex fitting frameworks are required^24^. In particular, measurement of rate constants governing vesicular efflux from different Golgi compartments as in ref. 27, under a calibrated increase in secretory cargo from the ER could potentially uncover saturation effects in vesicle budding and probe the existence of *K*_*sat*_, *m*_*sat*_.

Note that chemical interconversion in our model is also a highly simplified representation of the sequential processing of proteins as they pass through the Golgi^28^. Instead of explicitly incorporating the enzymes responsible for various biochemical modifications, we treat these modifications as Poisson processes occurring with specified rates, as in ref. 29. The rates should thus be viewed as effective or composite parameters, and can in principle be extracted by fitting the kinetics of ‘color change’ due to changing distribution of resident membrane proteins during maturation^17^,^18^. Despite its simplicity, we believe that such effective rates qualitatively capture the role of the interconversion process in shaping the macro organization of the Golgi.

The transport and chemical interconversion rules described above, can also be re-expressed as dynamical equations for the average “mass” of each chemical species at each site. Under suitable conditions, these equations can be solved to obtain the spatial profiles of the mass (abundance) of each species in steady state. From these profiles will emerge features such as ‘compartments’, for which we provide a precise definition below.

### Definition of a compartment

Given the finite spatiotemporal resolution of coarse-grained models, one needs to provide a consistent definition of a compartment, an aspect that is also pertinent for *in-vivo* imaging of Golgi compartments. As we will see, this is particularly germane to the VT model and its variants.

**Figure 2:**
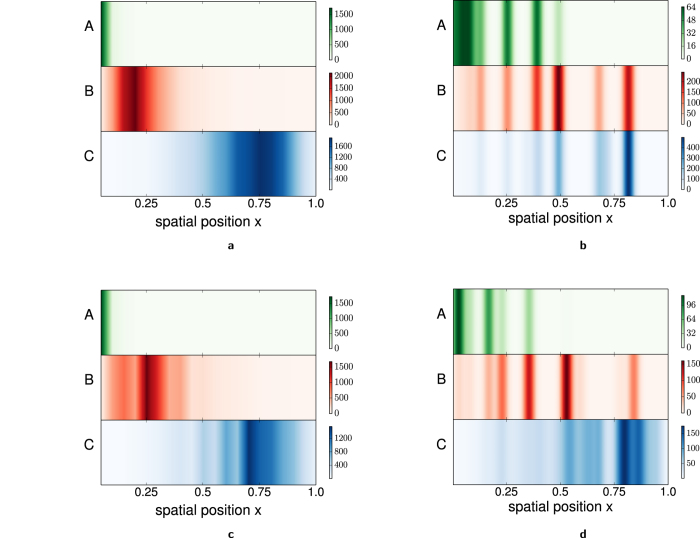
Typical steady state configurations for (**a**) pure VT (**b**) pure CP (**c**) VT-dominated transport with rare sub-cisternal movement, akin to the Cisternal Progenitor model and (**d**) CP-dominated transport with fission of single C particles. All configurations depicted in Gaussian-smoothed *compartment representation* where color intensity in each species channel indicates species mass (see LUT bar). (**a**) Pure VT model shows well-separated, pure (single-color) compartments while (**b**) pure CP model shows many mixed-color compartments with a gentle cis to trans gradient of each species. (**c**) VT-dominated transport: Mass profiles are qualitatively similar to the pure VT case, but with higher number of cisternal fragments between compartments. (**d**) CP-dominated transport: Sharp aggregates similar to those in the CP model are found in cis and medial regions of system, but disintegrate into smaller aggregates near the trans end. Parameters for (**a**): *a* = 1, *D* = 0, *w*_*A*_ = 0.125, *w*_*B*_ = 0.04375, *w*_*C*_ = 0.05462, *γ*_*A*_ = 0.5, *γ*_*B*_ = 0.6, *γ*_*C*_ = 0.66, *u* = 0.01125, *v* = 0.00194, *m*_*sat*_ = 200, *K*_*sat*_ = 14.14, *L* = 20; (**b**): *a* = 1, *D* = 0.00833, *α* = 1, *w*_*A*_ = *w*_*B*_ = *w*_*C*_ = 0, *u* = 0.025, *v* = 0.01166, *m*_*sat*_ = 200, *K*_*sat*_ = 14.14, *L* = 80. (**c**): *a* = 1, *D* = 0.000125, *α* = 0.3, *w*_*A*_ = 0.125, *w*_*B*_ = 0.0425, *w*_*C*_ = 0.05375, *γ*_*A*_ = 0.5, *γ*_*B*_ = 0.6, *γ*_*C*_ = 0.66 *u* = 0.01125, *v* = 0.001937, *m*_*sat*_ = 200, *K*_*sat*_ = 14.14, *L* = 20; (**d**): *a* = 1, *D* = 0.00833, *w*_*A*_ = *w*_*B*_ = 0, *w*_*C*_ = 0.8333, *γ*_*C*_ = 0.5, *u* = 0.025, *v* = 0.01662, *m*_*sat*_ = 200, *K*_*sat*_ = 14.14, *L* = 80.

To identify self-organized compartments we locate the maxima (concentration peaks) in the spatial profile of the total mass and note the locations on either side of the maximum where the mass drops to half its peak value. This half-width around maximum is taken to be the compartment size (and can span many lattice sites) while the sites between adjacent compartments constitute intercisternal regions. While defining dynamical moves (Fig. 1), however, we represent cisternal or sub-cisternal progression as the collective movement of the mass at a single site, rather than the movement of this many-site agglomerate. This leads to a consistent description in the CP limit where a compartment has a small width (spanning 1-2 sites). In the VT limit, where a typical compartment can span many sites (see Fig. 2a), the breakage, movement and fusion of sub-cisternal fragments from site to site within the compartment can be viewed as an intra-compartment remodeling process which eventually leads to the budding of a large fragment from the anterograde face of the compartment and its fusion with the next compartment.

### Units

Distances are shown in terms of the position variable *x*, which is scaled by *L*, estimated to be a few *μm*, depending on the cell type. Masses are defined as multiples of the unit mass, which is the typical mass of a vesicle or ‘particle’ breaking off from a cisterna (see Fig. 1); thus in our framework, mass of an aggregate denotes the number of vesicles it contains. Assuming vesicle diameter of 50–100 nm and typical surface area of a flattened 2D cisterna to be 6–30 *μm*^2 ^30,31 yields a rough estimate of 1000–10000 vesicles within each cisterna (neglecting fenestrae etc). A time unit (t.u.) is chosen such that the injection rate *a* is 1 vesicle/t.u.. Estimated influx of *a* ~ 1000–3000 vesicles/second^32^ imply that 1 t.u. is of the order of milliseconds. In the rest of the paper, all rates are specified in terms of (t.u.)^−1^.

## Results

### Structure formation in steady state

We now demonstrate how the interplay of various dynamical processes in the model gives rise to steady states configurations that mirror the spatial organization of Golgi cisternae. We first consider the two extreme limits of the model–the *pure VT* and the *pure CP* limits, and then explore variants around these limiting cases, with the aim of modeling ‘mixed’ traffic strategies.

### Structure formation with pure vesicular transport

The pure vesicular transport (VT) model is obtained by setting the aggregate movement rate *D* = 0. This allows us to solve for the average mass of each species at each site in steady state, using the techniques described in ref. 33 (see S2.1, SI for details). While we have explored several functional forms of *f*[*m*] using numerical simulations (S2.2, SI), we discuss below properties of structures generated using Eq. 1.

An example of compartment emergence in the pure VT limit for the three-species model with *A* → *B* → *C* conversion, is depicted in Fig. 2a. A typical snapshot shows that while *A* particles accumulate close to the cis end, *B* and *C* particles form distinct compartments in the cis to trans direction. Similarly, the four-species model exhibits steady states with A particles near the cis end and distinct B, C and D structures further on (see S2.3, SI). Self-organization of compartments in the pure VT limit is driven by three main effects, which we discuss below.

#### A. Spatial localization of compartments

The spatial segregation of chemical species and their localization in different regions, depends on (i) the ratio of chemical conversion rate to fission rates, *u*/*w*_*A*_, …, and (ii) the degree of directional bias in transport.

If the ratio *u*/*w*_*A*_ is very large, most A particles undergo an *A* → *B* conversion before they can travel into the interior, leading to an accumulation of B particles close to the source itself. Conversely, if *u*/*w*_*A*_ ≪ 1, B particles localize away from the source. The length scale governing the localization of C particles depends likewise on the ratio *v*/*w*_*B*_. The second determinant of the region of localization is the directional bias *γ*_*A*_, *γ*_*B*_, *γ*_*C*_ – particles with greater recycling to the source localize closer to the cis end, while those with less recycling form structures near the trans end. Thus, if the directional bias factors *γ*_*B*_ and *γ*_*C*_ are unequal, and the ratios *u*/*w*_*A*_ and *v*/*w*_*B*_ sufficiently different, the peak concentration of *B, C*, … occur at well-separated locations.

#### B. Morphologically distinct compartments

Spatially localized particles of a particular species form a large, morphologically distinct aggregate only if the flux-kernel *f*[*m*] attains a constant value at large *m* (as in Eq. 1), i.e., the outflux of vesicles from any aggregate saturates as aggregate size increases.

Sharp localization of B, C particles in different regions (as opposed to gentle gradients) is the key to obtaining spatially resolvable cisternae-like structures. To see how flux-kernels that saturate at large *m* lead to sharp localization, consider how the structures in Fig. 2a respond to a small increase in influx. An increase in influx increases the particle concentration or mass *m* in all regions of the system, which in turn causes the vesicular outflux (proportional to *f*[*m*]) to rise until a new flux balance is achieved. However, in regions of peak concentration, where *m* ≫ *m*_*sat*_ and the vesicular outflux *f*[*m*] is already in the saturation regime, the mass has to increase substantially in order to achieve the small increase in vesicular outflux required for steady state. Conversely, in the intercisternal regions, where *m* is small and *f*[*m*] is not in the saturation regime, a modest increase in mass is enough to achieve the requisite increase in vesicular outflux. Thus, with saturating flux kernels *f*[*m*] of the type in Eq. 1, the contrast in particle concentration between cisternal and intercisternal regions is naturally amplified when influx is high, leading to the emergence of sharply localized and well-defined compartments. In fact, if the influx is too high, the vesicular outflux may fail to balance the influx near the peaks, leading to runaway growth of mass, as elaborated in ref. 33 (also pointed out in ref. 29). The key to generating large but finite concentrations of A, B, C particles in specific regions, is to tune the influx rate *a* such that the system is in steady state, but *poised close to the transition point* to runaway growth. This causes the peaks in the average mass profile to become very sharp.

#### C. Robustness of compartments

There is a trade-off between the temporal stability of a compartment and how sharply localized it is; this can be optimized with a flux-kernel *f*[*m*] that increases for small *m* but saturates at large *m* (as in Eq. 1).

Stability of structures can be quantified using the ratio of the root mean square (rms) fluctuations Δ*m* to the mean mass 〈*m*〉 at any location. We measure this ratio for different functional forms of *f*[*m*] in our simulations (details in S2.2, SI), and find that aggregates are stable (Δ*m*/〈*m*〉 → 0 for large 〈*m*〉), if the fission rate and hence the flux-kernel *f*[*m*] increases with *m*. This ensures a stabilizing negative feedback–when the aggregate becomes too large, the number of particles breaking off from it increases and vice versa, thus restoring the aggregate to its average size. However, as discussed above, sharply localized A,B,C compartments emerge only if vesicular fluxes are relatively insensitive to aggregate size, i.e., for *f*[*m*] that saturates at large *m*. The best compromise between the competing requirements of stability and sharp localization is obtained with MM (or qualitatively similar) fission rates, as in Eq. 1.

Thus, spatially and chemically distinct compartments emerge in the pure VT limit, even in the *absence of a pre-existing template* if the three conditions above are satisfied (for a more rigorous discussion of the conditions, see S2.1, SI). A limitation of this mode of self-organization is that it requires *fine tuning* of parameters, and that structures may even undergo an instability, leading to runaway growth, in certain regions of parameter space. However, this instability is suppressed if we allow for a mixed transport strategy, in which vesicular transport is accompanied by rare sub-cisternal movement (see below).

### Structure formation with pure cisternal progression

The cisternal progression and maturation (CP) limit, obtained by setting *w*_*A*_ = *w*_*B*_ = *w*_*C*_ = 0 and *α* = 1, allows for anterograde movement of full aggregates at rate *D* (with aggregates fusing on encounter to form larger aggregates), in addition to the sequential *A* → *B* → *C* →  conversion of particles within each aggregate.

This system self-organizes into a state where the entire mass is concentrated in a relatively small number (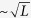) of aggregates (see Fig. 2b). The typical mass of an aggregate increases with increasing distance *x* from the (ER) source, while the probability of finding an aggregate decreases with *x*^34^. Thus, compartments become larger but sparser from cis to trans. Note that aggregate formation in the CP limit is a direct consequence of the stochastic nature of the model. If each aggregate were to move deterministically, as in the conveyor-belt-like scenario often invoked for cisternal progression^35^, then aggregates never encounter each other. Encounter and fusion of aggregates occurs because of stochastic fluctuation in the progression of different aggregates. The emergence of morphologically separate structures is thus a generic feature of this model, and occurs *without any fine tuning*.

The sequential chemical conversion of constituent particles further causes the system to develop biochemical polarity, with the composition of aggregates showing a gentle gradation from A-rich to B-rich to C-rich in the cis to trans direction. The length scales governing these chemical gradients depend on the interconversion rates and the function *f*[*m*]. System-wide gradients emerge for *u,v* ≪ *D*, i.e., for conversion rates that are much smaller than the rate of cisternal movement. A distinguishing feature of structure formation in the CP limit is the occurrence of a large number of mixed (two-color) compartments (Fig. 2b), which *change color* over time (movie S3, SI), much like the color change visualized for individual cisternae of the Golgi Apparatus in yeast^17^,^18^.

The movement of large aggregates through the system also results in distinctive dynamical properties: the total mass in the system undergoes *intermittent* time evolution^34^. We discuss later how this can be a revealing signature of cisternal passage. Most of these qualitative features of structure formation by CP appear robust upon changing transport rules, e.g., by making the aggregate movement rate *D* mass or chemistry dependent.

### Comparing structure formation in the two limits

While multiple, spatially and chemically distinct compartments emerge in both pure VT and CP models, there are significant differences between structures in the two limits that we discuss below.

First, structures generated in the pure VT model (with MM rates) are *stable* (position, size and composition vary little over time), though highly sensitive to changes in the rates of underlying processes. By contrast, in the pure CP model, structures are highly *dynamic*, with their positions, composition and even number showing significant variation over time. Monitoring the extent of variability of features such as the number of cisternae and the fluctuations in fluorescence from specific Golgi regions over time (see next section) using live-cell imaging could thus provide valuable clues to the mechanism of transport (to a limited extent this has been done in ref. 36).

Second, while cisternae in the VT limit are interconnected (Fig. 2a, also Fig. S4, SI), more discrete cisternae (with little or no mass in the intercisternal regions) arise in the CP limit (Fig. 2b). While the exact nature of this prediction may hinge on specifics of the model, the observation points towards an interesting correlation between the presence of inter-cisternal tubulation and the mode of intra-Golgi traffic.

Finally, the two limits also differ in the degree of spatial localization of individual chemical species. In the VT model, each species is *sharply localized* and not spread over multiple compartments (Fig. 2a). In the CP model, a typical snapshot (Fig. 2b) reveals many aggregates of mixed composition, with the relative abundance of each species exhibiting a gentle gradient across aggregates in the cis to trans direction. This distinction is fairly robust, suggesting possible experimental strategies aimed at exploring the connection between transport mechanisms and the spatial localization/delocalization of enzymes and markers, a connection that was also highlighted in ref. 37.

### Structure formation with mixed transport strategies

We now consider ‘mixed’ strategies of transport, which involve features of both the VT and the CP models to different extents^21^,^24^. This is also important from a stability perspective, since a mechanism for maintaining differentiated compartments must be robust to small variations in the model.

### Cisternal progenitor as a small deviation about pure vesicular transport

An undesirable property of structures formed in the pure VT limit is that they are quite sensitive to small variations in parameters and can even undergo an instability leading to runaway growth. This instability is ameliorated by allowing fragments of cisternae (containing a *finite fraction α* of cisternal mass) to bud off, move through the system and fuse with other compartments, as in the cisternal progenitor model^22^. In fact, using flux balance arguments, we can show that the inclusion of sub-cisternal movement at any *non-zero* rate *D* is sufficient to eliminate runaway growth, and ensure that aggregates always attain a constant average mass even under substantial changes in the rates of transport processes (see S3, SI).

Moreover, if the flux of cisternal fragments is much lower than vesicular fluxes between cisternae, then the well-separated *B* and *C* compartments typical of the pure VT model are preserved on an average (Fig. 2c). The movement of cisternal fragments does result in significant stochastic fluctuations (Δ*m*/*m* ~ *α*) about the average mass profile over time scales proportional to 1/*D*. These fluctuations, are however, small as long as *α* is small. Thus, the addition of a cisternal-progenitor-like process at a small rate to vesicular transport appears to be a very reliable control mechanism, meeting all the desiderata of organelle morphogenesis. In the rest of the paper, we refer to this strategy as the *VT-dominated* case.

### Adding some vesicular exchange to pure cisternal progression

Consider now a scenario where the bulk of transport is via cisternal movement, but with vesicular fission and exchange between cisternae also occurring at a small rate. As elaborated in ref. 34, the system continues to support large aggregates as long as the fission rate is small (≪*a*/*D*) and vesicular fluxes are independent of the mass of large parent aggregates. However, an interesting situation arises when different species have very different fission rates, e.g., if *w*_*C*_ is large, while *w*_*A*_ and *w*_*B*_ are small. In this case, large aggregates form close to the cis end but disintegrate into smaller fragments near the trans end, giving rise to configurations (Fig. 2d) that bear a striking resemblance to the usual picture of cisternal progression. In the rest of the paper, we refer to this scenario as the *CP-dominated* case.

Our analysis of mixed transport scenarios suggests that these are viable mechanisms for the formation and maintenance of differentiated cisternae, as long as one of the transport processes (vesicular or cisternal movement) is dominant, and the alternative process acts at a much smaller rate as a secondary track for traffic. If both processes occur with comparable rates, then large structures tend to dissipate, and the system homogenizes.

### Dynamical measurements can discriminate between transport models

Below we discuss how experimental approaches that monitor dynamics of the Golgi in steady state or dynamics of recovery following perturbations from steady state, can be used to discriminate between various models. A dynamics-based approach was also explored in ref. 35, where the temporal pattern of fluorescence decay in iFRAP experiments was used to test several hypotheses about molecular trafficking in the Golgi (see ref. 29 for a quantitative analysis, also S5.1, SI). Here, we propose several novel dynamical measurements that can provide a window into traffic processes.

### Steady state dynamics of the Golgi

**Figure 3:**
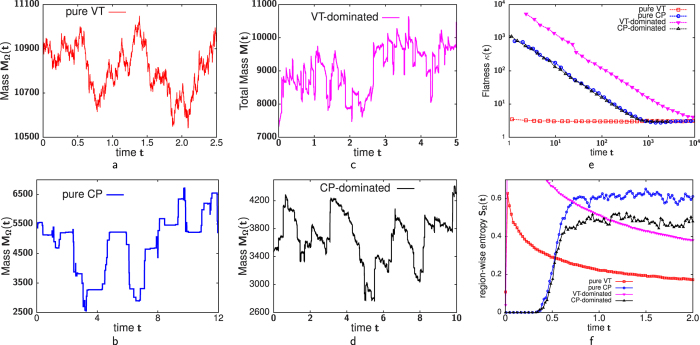
(**a**–**d**) Mass *M*_Ω_(*t*) in the region (0.25 < *x* ≤ 0.75 ∈ Ω) vs. time *t* in steady state for (**a**) pure VT (**b**) pure CP (**c**) VT-dominated model (with sub-cisternal movement) (**d**) CP-dominated model (with fission of C-type vesicles). Scale of x-axis is 1 ≡ 10^5^ t.u. for (**a**,**c**), and 1 ≡ 10^3^ t.u. for (**b**,**d**). Cisternal or sub-cisternal movement in cases (**b**–**d**) leads to sharp changes in *M(t*). (**e**) Flatness *κ(t*) vs. *t* on a log-log plot. Sharp changes in *M(t*) in cases (**b**–**d**) cause divergence of *κ(t*) at small *t*. No such divergence with pure VT. The time scale over which *κ(t*) diverges is roughly the time interval between successive cisternal passage events, and is very large for the VT-dominated case where sub-cisternal movement occurs rarely. (**f**) Dynamics during de novo biogenesis: Compositional entropy *S*_Ω_(*t*) in the region (0.5 < *x* ≤ 0.75 ∈ Ω), averaged over 100 realizations, decreases with *t* for vesicle-dominated transport, and increases with *t* for cisterna-dominated transport. Scale of x-axis is 1 ≡ 10^5^ t.u. in the pure VT and VT-dominated cases and 1 ≡ 10^4^ t.u. in the pure CP and CP-dominated cases. All simulation parameters same as in Fig. 2.

Distinctive signatures of the underlying traffic mechanism can be observed in the time series and fluctuation statistics of the “mass” *M*_Ω_ in (any) macroscopic region Ω of the system. Figure 3a–d depicts *M*_Ω_(*t*) vs. *t* for the central region Ω:0.25 <*x *≤ 0.75 for the different models (see also SI movies (S1,S2,S3,S4)). While the time series in the pure VT model has a smoothly varying, nearly Brownian character (Fig. 3a), in the pure CP model, it is ‘intermittent’ and displays sharp changes, corresponding to the entry and exit of large aggregates from the region Ω (Fig. 3b). For the mixed transport, i.e., the VT-dominated and the CP-dominated models, the time series show stretches of smooth variation, interspersed with an occasional sharp rise or fall (Fig. 3d).

These observed differences can be quantified in terms of *dynamical structure functions S*_*n*_(*t*) = 〈[*M*_Ω_(*t*) − *M*_Ω_(0)]^*n*^〉 and their ratios, in particular the ‘flatness’^34^,


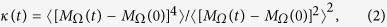


which is essentially a time-dependent kurtosis, the average 〈…〉 being over several measurements of *M(t*). If *M(t*) shows variation on two very different scales, e.g., zero or small changes from vesicular fluxes and very large changes from cisternal or sub-cisternal passage, then *κ(t*) is expected to show a divergence at small *t*, arising from the large changes^34^,^38^. We find that *κ(t*) diverges as *t* → 0 for all the three cases–pure CP, CP-dominated, VT-dominated–n which there is appreciable cisternal or sub-cisternal movement, but remains ‘flat’ in the pure VT case (Fig. 3e). To further distinguish between the CP-dominated and VT-dominated scenarios, we monitor the *timescale* over which flatness diverges–this is just the typical time period between successive cisternal passage events, and is much larger for the VT-dominated limit, as compared to the CP-dominated limits (Fig. 3e). More generally, if the frequency of cisternal passage events (as inferred from flatness measurements) is consistent with the observed rates of cargo transit, then this points towards a cisterna-dominated mode of traffic, whereas if the frequency is much less, then this would suggest that cisternal movement is only a secondary transport mechanism.

Statistics such as flatness can be extracted by monitoring the time series of fluorescence intensity of Golgi markers, and should be accessible in recent dynamical measurements at sub-Golgi resolution^39^. It may also be instructive to do this measurement separately for the cis, medial and trans regions of the Golgi, to test whether intensity fluctuations from cis and medial Golgi have qualitatively different statistics compared to fluctuations from the trans Golgi, where transport is dominated by vesicles and fragments of the disintegrating cisterna.

### Dynamics of de novo biogenesis

To study the dynamics of de novo biogenesis, we start with an initial condition in which the system is completely empty (corresponding to resorption of cargo molecules and Golgi-resident enzymes into the ER, for instance after Brefeldin treatment). We then restart all the elementary processes that define the dynamics of the model (this corresponds to a washout of Brefeldin in regeneration experiments^40^), at the chosen rates, and monitor how the *A, B, C* mass profiles recover in time *t*. Our simulations show that these profiles evolve quite differently, depending on whether vesicular or cisternal movement is the dominant mode of transport (movies (S5 and S6)).

A clear signature of the transport process appears in the region-wise compositional ‘entropy’, defined as:


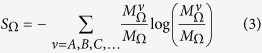


where 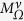 is the mass of species *ν* in the region Ω, and 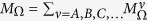. The quantity *S*_Ω_ is a measure of the compositional heterogeneity of a compartment, being high for compartments with multiple species of particles, and low for compartments with predominantly one constituent type. The region Ω chosen for measurement must be of appropriate size–neither so large that it spans multiple compartments, nor so small that the *M*_Ω_(*t*) signal is dominated by noise. When cisternal movement is the predominant mode of transport, the region-wise entropy *S*_Ω_(*t*) increases with *t* as the system regenerates, in contrast to the pure VT and VT-dominated limits, where *S*_Ω_(*t*) is a decreasing function of *t*, except for an initial transient rise (Fig. 3f).

In the CP limit, rising entropy in the cis compartments is a direct consequence of maturation–the early aggregate is purely of type A, but changes from A to AB to ABC to BC over time, thus increasing the entropy in this region. Near the trans end, maturation has the opposite effect, shifting the composition of aggregates from BC towards pure C and reducing the entropy. But this is countered by the entry of mixed BC compartments and exit of concentrated C compartments, resulting in a net increase in compositional entropy even in the trans compartments, on average. Thus, *S*_Ω_(*t*) rises during de novo formation with CP due to the generation and movement of *mixed* compartments. From the above it also follows that fast chemical conversion leads to fast growth of entropy in the cis region, while in the trans compartments, where maturation tends to reduce compositional heterogeneity, higher *u,v* have the opposite effect. Further, slow cisternal passage (low *D*) results in slower regeneration of the structures and longer timescales of entropy change in all regions.

In the VT limit, new *C* compartments are not generated by the maturation of *B* compartments via mixed *BC* intermediates, but instead arise due to the preferential localization of *B* and *C* particles in different regions. The long period of decreasing entropy thus corresponds to growth of *B* and *C* particles in different regions and the increase in the ‘purity’ of the two compartments. As in the CP model, the time scale over which entropy is lost may increase or decrease with interconversion rates, depending on the region under consideration. In general, low influx *a* results in a weaker and shorter buildup of *B, C*,… concentration in different regions, and hence a faster rate of entropy loss.

In the SI (sec. S5.2), we also describe the dynamics of reconstitution (from an initial state where all molecules and markers are distributed in the Golgi region in a uniform, unpolarized manner) and discuss how this differs from de novo formation.

### Perturbation and response

**Figure 4:**
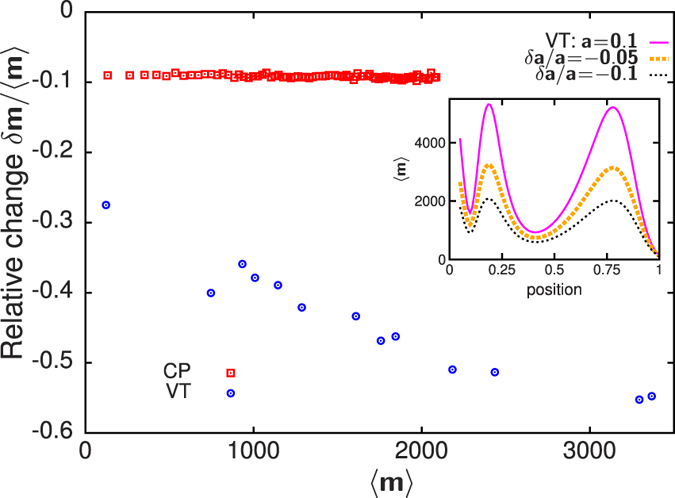
Relative change *δm*/〈*m*〉 (at a location with average local mass 〈*m*〉) when influx *a* falls by 10%. In the CP limit, *δm*/〈*m*〉 is independent of 〈*m*〉, while in the VT limit, |*δm*|/〈*m*〉 increases with 〈*m*〉: larger masses show a stronger proportionate change than smaller masses. Inset: In the VT limit, mass profiles change shape i.e., become much less peaked as *a* is reduced. Parameters for (i) VT: *a* = 1, *D* = 0, *w*_*A*_ = 0.11364, *w*_*B*_ = 0.03977, *w*_*C*_ = 0.04966, *γ*_*A*_ = 0.5, *γ*_*B*_ = 0.6, *γ*_*C*_ = 0.66, *u* = 0.01023, *v* = 0.00176, *m*_*sat*_ = 200, *K*_*sat*_ = 14.14, *L* = 20; (ii) CP: *a* = 1, *D* = 0.00757, *α* = 1, *w*_*A*_ = *w*_*B*_ = *w*_*C*_ = 0, *u* = 0.02273, *v* = 0.01061, *m*_*sat*_ = 200, *K*_*sat*_ = 14.14, *L* = 80.

We now consider how the steady state structures in the various models respond to perturbations such as (i) variation in influx and (ii) exit block at the trans end. (i) Change in influx: The extent of cargo load has been posited as an important determinant of Golgi size^41^. How does the average mass 〈*m*〉 at any location within the Golgi respond when the ER to Golgi influx shifts from *a* to *a* ± *δa*? Figure 4 shows the relative change *δm*/〈*m*〉 as a function of 〈*m*〉 for the pure VT and pure CP models, for *δa*/*a* = −0.1. In the CP model, *δm*/〈*m*〉 is independent of 〈*m*〉, indicating that all cisternae, whether large or small, show the same proportionate reduction in volume on an average. By contrast, in the VT model, |*δm*|/〈*m*〉 increases with 〈*m*〉. Thus, the relative change is maximum where local mass is highest and minimum for regions with low mass, resulting in a new mass profile that is less peaked (inset, Fig. 4). The strong, shape-altering response of the mass profile in the VT limit is a direct consequence of the flux-kernel *f*[*m*] saturating at large *m*. Large aggregates, which operate in this saturation regime, must undergo a substantial change in size in order to produce the small changes in vesicular outflux required to balance the altered influx. Note that while cisternal sizes respond to altered influx in both VT and CP limits, it may be difficult to distinguish a systematic change from large stochastic fluctuations in the pure CP limit without sufficient statistical averaging (see also movies (S9,S10,S11,S12)).

(ii) Exit block: Golgi transport is highly temperature-sensitive, with budding and exit from the trans Golgi suppressed at low temperatures (~20 °C), resulting in lateral expansion of the trans compartments^42^. We simulate the 20 °C block by stopping the exit of particles from the right boundary, and find that particles pile up indefinitely at the exit site in both the CP and VT limits (see movies (S13 and S14)). This uncontrolled pile-up is unrealistic for the Golgi, but raises the interesting question of how the size of trans compartments is regulated when exit is blocked–whether there is an alternative pathway for the degradation of the trans Golgi or some feedback mechanism that eventually suppresses influx from the ER.

## Discussion

In this paper, we have defined and studied a general but minimal stochastic model of a system of compartments within a trafficking pathway. While we have chosen to focus on the Golgi apparatus, the model is equally relevant to the endosomal system. Our model is broad enough to survey several known local dynamical rules of transport and chemical transformation, and encompasses vesicular transport, cisternal progression, cisternal progenitor and its variants. We identified conditions under which this nonequilibrium open system self-organizes into Golgi-like structures, i.e., into multiple, spatially resolvable aggregates with a position-dependent chemical composition. We emphasize that the models studied here perforce have a many-body character arising from interactions between the constituent particles, this implies that *local* changes in parameter values can have *nonlocal* phenotypic consequences.

A unique feature of our model is that it explicitly incorporates spatial degrees of freedom, by assuming a 1D lattice of fixed size *L*. While this does impose an extraneous length scale on the system, the length scales that determine where different species localize or how far from the ER different compartments form, are emergent length scales which depend on the ratio of the conversion rate to the vesicle or cisternal movement rates as well as the retrograde/anterograde transport asymmetry. In fact, the exact position of *L* only affects the spatial distribution the ultimate processed product (*C* here) which is taken out at the PM.

In the context of vesicular transport, we have emphasized (as has^29^) the importance of the MM-form of the flux kernel *f*[*m*] for fission and chemical conversion processes, which shows saturation at large *m*. Experimentally probing saturation effects in vesicular fluxes as compartment size is varied, can be an interesting direction for future work, and may also have broad implications for formation and maintenance of sub-cellular structures.

Significantly, our model generates chemically polarized compartments even in the absence of directed and specific fusion, an important ingredient of previous models^7^,^29^. In these models, directed fusion essentially results in autocatalytic accumulation of a particular chemical species in a particular compartment. This appears to be necessary for formation of non-identical compartments in a situation where the dynamics is completely symmetric^7^. However, our model suggests that in the scenario where there is an inherent asymmetry or directionality, as in the case of the Golgi apparatus, specific fusion may be less central to the maintenance of compartments with distinct chemical identities.

A limitation of this study is that it does not explicitly take into account the role of enzymes in the processing of cargo molecules in the Golgi, and the complex dynamics of the enzymes themselves. Thus, extending the present model to include enzymes as separate entities would lead to a more realistic model, the parameters of which could also be connected to experimentally relevant quantities. However, the increase in the number of parameters also makes it difficult to analyze such an extended model and extract the relevant regions of parameter space. An abstract model of the sort studied in this paper, is more suited to gain insight into the qualitative effects at play in the system.

At a philosophical level, our work touches upon the question of whether and how the Golgi can form de novo (for example, after treatment with reagents that cause Golgi cisternae to disintegrate). We show that a system with “Golgi-like traffic processes” has the ability to self-organize into morphological and chemically distinct structures that share many qualitative features of Golgi cisternae. This self-organization happens even in the limit corresponding to vesicular transport, in the absence of any template. In essence, the molecular machinery associated with the stated dynamical rules of the minimal models are the only ones necessary for Golgi inheritance. Indeed, this and many of our mathematically derived conclusions find resonance in ideas articulated before, especially in ref. 4. An important conclusion of our analysis is that the transport mechanism can qualitatively impact structural features of the cisternae such as inter-cisternal tubulations or the degree of localization of Golgi-resident enzymes. Such correlations between transport and morphology could be explored by doing a comparative study across different cells. Finally, we propose that fluctuation statistics in fluorescence experiments could be a powerful probe of Golgi dynamics, with sporadic and large fluctuations, in particular, revealing cisternal passage events.

## Additional Information

**How to cite this article**: Sachdeva, H. *et al*. Nonequilibrium description of de novo biogenesis and transport through Golgi-like cisternae. *Sci. Rep.* **6**, 38840; doi: 10.1038/srep38840 (2016).

**Publisher's note:** Springer Nature remains neutral with regard to jurisdictional claims in published maps and institutional affiliations.

## Supplementary Material

Supplementary Material

Supplementary Movie S1

Supplementary Movie S2

Supplementary Movie S3

Supplementary Movie S4

Supplementary Movie S5

Supplementary Movie S6

Supplementary Movie S7

Supplementary Movie S8

Supplementary Movie S9

Supplementary Movie S10

Supplementary Movie S11

Supplementary Movie S12

Supplementary Movie S13

Supplementary Movie S14
