# Supplementary material for: Nonequilibrium description of de novo biogenesis and transport through Golgi-like cisternae
Source: Sci Rep. 2016 Dec 19;6:38840. doi: 10.1038/srep38840 (PMC5171829; doi:10.1038/srep38840)
Supplement: Supplementary Material [file srep38840-s1.pdf]

Supplementary Online Material for

Nonequilibrium description of de novo biogenesis and transport through Golgi-like cisternae

Himani Sachdeva[1,2], Mustansir Barma[1,3] and Madan Rao[4,5]

[1]Department of Theoretical Physics, Tata Institute of Fundamental Research, Homi Bhabha Road, Mumbai 400005, India

[2]Institute of Science and Technology, Am Campus 1, Klosterneuburg A-3400, Austria

[3]TIFR Centre for Interdisciplinary Sciences, 21 Brundavan Colony, Narsingi, Hyderabad 500075, India

[4]Raman Research Institute, C.V. Raman Avenue, Bangalore 560080, India

[5]Simons Centre for the Study of Living Machines, National Centre for Biological Sciences (TIFR), Bellary Road, Bangalore 560065, India

Contents

S1 Model 1

S1.1 Detailed Description . . . 1

S1.2 Rationalizing the model . . . 2

S1.3 Dynamical equations for mass . . . 3

S2 Analysis of the pure Vesicular Transport (VT) case 3

S2.1 Insights from the analytical solution . . . 3

S2.2 Structure formation with different types of flux kernels $f[m]$ . . . 6

S2.3 Pure VT model with higher number of species . . . 9

S2.4 Effect of homotypic fusion . . . 10

S3 VT-dominated case: Effect of (sub-)cisternal movement 10

S4 Typical steady state configurations in aggregate representation 11

S5 Dynamical measurements 11

S5.1 Dynamics of (fluorescently) tagged particles . . . 11

S5.2 Dynamics of reconstitution after disassembly . . . 12

S6 Captions for Supplementary Movies (S1)-(S14) 14

S1 Model

S1.1 Detailed Description

The model is a 1D lattice model with three species, ‘A’, ‘B’ and ‘C’ of particles (see Fig.1 of main paper for a schematic). It incorporates the main processes involved in Golgi dynamics as stochastic moves (listed below) that occur with a constant rate per unit time.

i. *Influx* of single particles of type A at the leftmost site (ER) with rate a.

ii. *Chemical conversion* of an A particle to a B particle at site i with rate $uf[m_i^A]$ that depends on the "mass" (number of particles) m_i^A of A through the flux-kernel $f[m_i^A]$. Similarly, a $B\to C$ conversion can occur at site i with rate $vf[m_i^B]$, and so on [1].

iii. *Fission, movement and fusion of single particles* with species-dependent rates: An A particle fissions from site i, moves in the anterograde (or retrograde) direction with rate $w_A f\left[m_i^A\right]$ (or $w'_A f\left[m_i^A\right]$), and fuses with the mass on the neighboring site. Similarly a B (or C) particle fissions and moves forward with rates $w_B f\left[m_i^B\right]$ (or $w_C f\left[m_i^C\right]$) and backward with rates $w'_B f\left[m_i^B\right]$ (or $w'_C f\left[m_i^C\right]$).

iv. *Breakage of a finite fraction (α) of an aggregate and movement* in the anterograde direction with rate D (α=1 corresponds to cisternal progression, and α<1 to sub-cisternal movement.).

v. *Exit of full aggregates or single particles from boundaries*, with the same rates as those for movement in the bulk.

The vectorial nature of transport is modeled through the asymmetry in the anterograde/retrograde particle movement rates, and is parametrized as: $w_A\to\gamma_A w_A$, $w'_A\to(1-\gamma_A)\,w_A$, $w_B\to\gamma_B w_B$, $w'_B\to(1-\gamma_B)\,w_B$ and $w_C\to\gamma_C w_C$, $w'_C\to(1-\gamma_C)\,w_C$ (see Fig. 1 of main paper). The asymmetry factors γ_A, γ_B, γ_C for the three species can be different, representing differential degrees of recycling of $A, B, C, \ldots$ particles to the ER (a generalization of [1]).

The parameter space of this model is quite large, encompassing the injection rate a, the interconversion rates $u, v, \ldots$ the fission rates $w_A, w_B, w_C, \ldots$ and the corresponding asymmetry factors $\gamma_A, \gamma_B, \gamma_C, \ldots$, the aggregate movement rate D, the breakage fraction α, and finally the form of the function f itself.

S1.2 Rationalizing the model

Below we comment in some detail on various aspects of the model and also highlight its strengths and weaknesses as a model of Golgi biogenesis.

1. Representing the three-dimensional Golgi by a one-dimensional model:
   Our model is a spatial model that explicitly incorporates distance from the cis end as a relevant variable. In the interest of analytical tractability, we include only one spatial dimension corresponding to the cis to trans direction, which is the main direction of molecular traffic, and also the axis along which the Golgi exhibits biochemical polarity. The 1D model can thus be considered an effective model obtained by integrating over the two directions perpendicular to this cis to trans direction. However, a 1D model of this sort cannot address questions related to the shape of individual cisternae or if trafficking pathways are branched.

2. A,B,C particles represent molecules in different stages of processing in the Golgi:
   In constructing this model, we imagine an A particle to be the equivalent of the unprocessed protein molecules arriving at the cis-Golgi from the ER, and particles of type B, C, D as representing proteins in different (successive) stages of processing. Likewise, an aggregate which is primarily of type B is analogous to a cisterna with a large fraction of semi-processed molecules in an early stage of processing. Thus, in our model, the processing stages 'A', 'B', 'C' of the primary constituent molecules of a cisterna define the chemical identity of the cisterna.

3. Modeling enzyme-mediated cargo modifications as simple Poisson processes:
   The $A\to B$ and $B\to C$ conversions which we have treated as Poisson rate processes in the model are catalyzed by enzymes in the real system. A more realistic model of the Golgi could include both A, B, C 'cargo species' and E_A, E_B 'enzyme species', with specific enzymes having an affinity for specific cargo types. This kind of a detailed enzyme plus cargo model would allow us to model feedbacks

[1] In principle, the reactions $B\to A$ and $C\to B$ can also be included, but to limit the number of parameters, we do not allow for these. The basic features of the model are not altered in the presence of these reverse reactions, as long as there is a net forward rate of reaction.

wherein the distribution of B particles influences the distribution of the corresponding E_B enzymes, and is in turn influenced by E_B molecules which activate the $B \to C$ conversion. Thus, in general, we expect self-organization in this extended model to be more complex. Nevertheless, the effective rate-based model studied in this paper incorporates the sequential interconversion process using just a few parameters, and has the advantage that it is more amenable to analysis than a complex model. Moreover, as a *spatial* model that incorporates both chemical and transport processes in the Golgi, it provides a valuable framework for constructing a more detailed model with enzymes.

4. Model parameters are composites of many biophysical rates:
The various particle exchange and interconversion rates in our model are effective or composite rates. For example, the particle movement rate combines both the rate at which a vesicle buds from an aggregate, as well as the rate at which it fuses with the next aggregate. Similarly, the $B \to C$ modification rate is determined jointly by the rates of attachment and detachment of enzyme molecules with B molecules, the concentration of the enzyme molecules, the reaction rate between the enzyme and the B molecule etc. . Because of the composite nature of the rates, it is not straightforward to find the corresponding experimentally measurable parameter. However, a model of this sort with a relatively small number of composite parameters allows for an economical description of the system, which makes it easier to identify and elucidate the qualitative effects at play in the system.

S1.3 Dynamical equations for mass

The following dynamical equations describe the time evolution of the average mass (number of particles) of each species at each site i in the lattice, in accordance with the elementary moves allowed in the model:

$$\frac{\partial \langle m_i^A(t)\rangle}{\partial t} = w_A \left\{ \gamma_A \langle f[m_{i-1}^A]\rangle + (1-\gamma_A)\langle f[m_{i+1}^A]\rangle - \langle f[m_i^A]\rangle \right\} + \alpha D \left\{ \langle m_{i-1}^A\rangle - \langle m_i^A\rangle \right\} - u\langle f[m_i^A]\rangle \tag{S1a}$$

$$\frac{\partial \langle m_i^B(t)\rangle}{\partial t} = w_B \left\{ \gamma_B \langle f[m_{i-1}^B]\rangle + (1-\gamma_B)\langle f[m_{i+1}^B]\rangle - \langle f[m_i^B]\rangle \right\} + \alpha D \left\{ \langle m_{i-1}^B\rangle - \langle m_i^B\rangle \right\} + u\langle f[m_i^A]\rangle - k\langle f[m_i^B]\rangle \tag{S1b}$$

$$\frac{\partial \langle m_i^C(t)\rangle}{\partial t} = w_C \left\{ \gamma_C \langle f[m_{i-1}^C]\rangle + (1-\gamma_C)\langle f[m_{i+1}^C]\rangle - \langle f[m_i^C]\rangle \right\} + \alpha D \left\{ \langle m_{i-1}^C\rangle - \langle m_i^C\rangle \right\} + k\langle f[m_i^B]\rangle \tag{S1c}$$

$$w_A\gamma_A\langle f[m_0^A]\rangle = a; \quad \langle f[m_0^B]\rangle = \langle f[m_0^C]\rangle = 0; \quad \langle f[m_{L+1}^A]\rangle = \langle f[m_{L+1}^B]\rangle = \langle f[m_{L+1}^C]\rangle = 0 \tag{S1d}$$

where $\langle \ldots \rangle$ indicates averaging over ensembles. The mass of each species at each site changes due to single particle exchange and aggregate movement between neighboring sites, as well as interconversion at any given site. If the system attains steady state, then the time derivatives in eqs. (S1a)-(S1c) can be set to zero, so that the equations take the form of flux-balance conditions for each site.

S2 Analysis of the pure Vesicular Transport (VT) case

S2.1 Insights from the analytical solution

In the pure VT limit (with $D=0$), eq. (S1) can be solved in the steady state, by setting time derivatives to zero and taking a continuum limit $i/L \to x$ in space, which transforms the equations into a set of coupled, second-order ODEs. Solving these ODEs yields $\langle f[m^A(x)]\rangle$, $\langle f[m^B(x)]\rangle$ and $\langle f[m^C(x)]\rangle$ as a function of x. For the three-species model, the solutions are quite involved, and it is more convenient to solve eq. (S1) numerically. Figure S1(a) shows numerically obtained solutions for a particular choice of parameters. The mass profiles $\langle m^A(x)\rangle$, $\langle m^B(x)\rangle$ and $\langle m^C(x)\rangle$ can be approximately derived from this solution by assuming that the mass m at any instant is close to its average value $\langle m\rangle$, so that $\langle f[m]\rangle \sim f[\langle m\rangle]$. For instance, if

$f[m^Z]$ is of the Michelis-Menten (MM) form [eq. (S2a)] (where Z can denote any of A, B, C), then the mass profiles can be approximated using eq. (S2b) [see also fig. S1].

$$f[m^Z] = \frac{K_{sat}\sqrt{m^Z}}{\sqrt{m^Z} + \sqrt{m_{sat}}} \tag{S2a}$$

$$\langle m^Z \rangle \sim \left(\frac{\sqrt{m_{sat}}\langle f[m^Z]\rangle}{K_{sat} - \langle f[m^Z]\rangle} \right)^2 \tag{S2b}$$

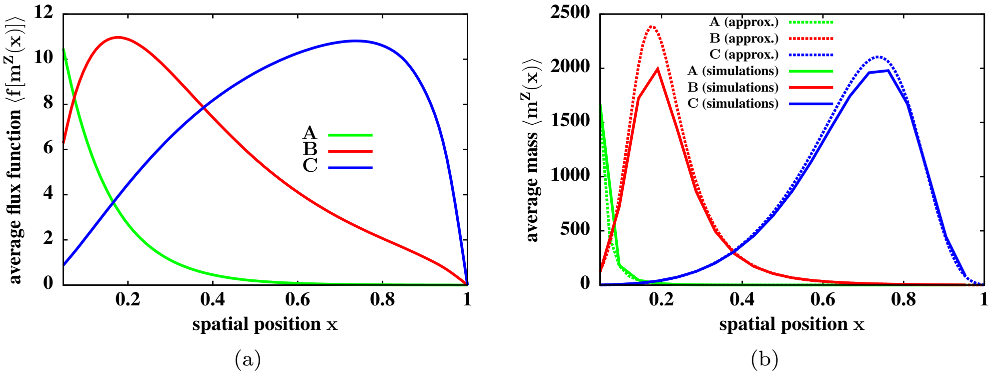

Figure S1: (a) Equation (S1) can be solved numerically in the continuum limit to obtain $\langle f[m^A(x)]\rangle$, $\langle f[m^B(x)]\rangle$ and $\langle f[m^C(x)]\rangle$ vs. x (here, for $a{=}1$, $D{=}0$, $w_A{=}0.125$, $w_B{=}0.04375$, $w_C{=}0.05462$, $\gamma_A{=}0.5$, $\gamma_B{=}0.6$, $\gamma_C{=}0.66$, $u{=}0.01125$, $v{=}0.00194$, $m_{sat}{=}200$, $K_{sat}{=}14.14$). (b) The approximate $\langle m^A(x)\rangle$, $\langle m^B(x)\rangle$, $\langle m^C(x)\rangle$ profiles (dashed lines) derived using eq. (S2b) are reasonably close to the actual mass profiles (solid lines) from simulations.

The key characteristics of the mass profiles can be illustrated using a two-species version of the model with 'A' and 'B' particles that undergo $A{\to}B$ conversion, and move with the same directional bias $\gamma_A{=}\gamma_B{=}\gamma$. In this case, the analytical steady state solution for $\langle f[m^A(x)]\rangle$ and $\langle f[m^B(x)]\rangle$ is relatively simple [1]:

$$\langle f[m^A(x)]\rangle = \frac{a}{w_A\gamma}\exp(\tilde{\xi}x)\left(\frac{\sinh\left(\sqrt{\tilde{\eta}+\tilde{\xi}^2}(1-x)\right)}{\sinh\left(\sqrt{\tilde{\eta}+\tilde{\xi}^2}\right)}\right), \qquad \tilde{\eta} = \frac{2uL^2}{w_A}, \quad \tilde{\xi} = (2\gamma-1)L \tag{S3a}$$

$$\langle f[m^B(x)]\rangle = \frac{a}{w_B\gamma}\left[\frac{1-\exp(-2\tilde{\xi}(1-x))}{1-\exp(-2\tilde{\xi})} - \exp(\tilde{\xi}x)\left(\frac{\sinh\left(\sqrt{\tilde{\eta}+\tilde{\xi}^2}(1-x)\right)}{\sinh\left(\sqrt{\tilde{\eta}+\tilde{\xi}^2}\right)}\right)\right] \tag{S3b}$$

Below, we discuss some salient features of this solution:

1. *Stationary state vs. runaway growth*: Consider a flux kernel $f[m]$ of the sort in eq. (S2a), which saturates to a constant value K_{sat} at large m. The average $\langle f[m^A(x)]\rangle$ and $\langle f[m^B(x)]\rangle$ functions, as given by eq. (S3), must be less than this maximal value K_{sat} everywhere, for eq. (S3) to be the correct steady state solution. For some parameters, however, unphysical solutions arise with $\langle f[m^A(x)]\rangle$ and/or $\langle f[m^B(x)]\rangle$ exceeding K_{sat}. These parameters are precisely those for which the assumption of steady state ($d\langle m\rangle/dt{=}0$) breaks down; instead, the mass undergoes runaway growth: $d\langle m\rangle/dt{>}0$ at all times (for details, see [1]). Figure S2 depicts a phase diagram for the 2-species model with $\gamma{=}1/2$, where

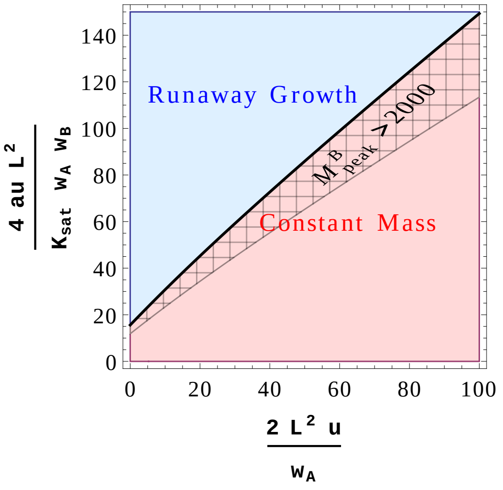

Figure S2: Phase diagram for the simplified two-species model with $\gamma_A = \gamma_B = 1/2$, $2a/w_A < 1$ and $f[m]$ of the MM type. Parameter combinations in the blue region lead to runaway growth of B particles; parameters in red region correspond to steady state, and the bold line is the boundary between the two phases. For parameters in the shaded red region, $\langle m^B \rangle > 2000$ at the peak of the B profile (as deduced from the approximation in eq. (S2b)).

parameter combinations in the blue and red regions lead to runaway growth of B particles and steady mass of B particles respectively. Physically, runaway growth arises because the outgoing vesicular fluxes at a site saturates if the mass at the site is much higher than m_{sat}. Thus, at sufficiently high rates of influx, the outgoing flux from a location may fail to balance the incoming flux, leading to runaway growth. A more detailed analysis shows that typically, runaway growth occurs only in specific regions of the system, even as the rest of the system attains steady state [1].

2. *Obtaining sharp peaks with large but finite mass:* The transition from steady state to runaway growth at a particular site is associated with divergence of the average mass at that site (see eq. (S2b), where the mean mass $\langle m \rangle$ diverges as $\langle f[m] \rangle \to K_{sat}$). Thus, by tuning the system to be close to the phase boundary between steady state and runaway growth (represented in fig. S2 by the solid black line), the peak concentration of particles for any species can be made very high. (For instance, see fig. S2, where parameter combinations in the shaded region result in a steady state in which the peak concentration of B is greater than 2000 particles at a site). Moreover, even though the $\langle f[m] \rangle$ profile is relatively smooth, the $\langle m \rangle$ profile becomes very sharply peaked when the system is poised close to the phase boundary, leading to a sharply localized B-rich region.

3. *Tuning the locations of mass peaks:* The location of the peak of any mass profile (here the $\langle m^B(x) \rangle$ profile) is determined by two length scales, l_c and l_t, which govern the rise and fall of the $\langle f[m^B(x)] \rangle$ and $\langle m^B(x) \rangle$ profiles near the cis and trans ends respectively. For the two-species case, eq. (S3) can be analyzed (see [1]) to show that (i) the length scale l_c is small if the ratio u/w_A is large: For high values of u/w_A, most A particles undergo an $A \to B$ conversion before they can travel into the bulk, so that the concentration of B particles rises steeply close to the source itself, leading to a small l_c (ii) l_c is large if the asymmetry factor γ is high: As γ increases, the proportion of B particles traveling in the anterograde direction rises, shifting the region of high B concentration in this direction, thus leading to large l_c. (iii) The length scale l_t, which governs the spatial variation of $\langle f[m^B(x)] \rangle$ near the trans end, only depends on the asymmetry factor γ, and is large when γ is small: The presence of the particle 'sink' at the trans end lowers particle concentration in this region. If there is extensive recycling or movement of particles in the retrograde direction (corresponding to γ close to 1/2), then the effect of the sink is 'transmitted' in this direction, lowering the local mass farther away from the

trans end, resulting in large l_t. The locations of the maxima of mass profiles in the 3-species model are also determined in a similar manner by the ratio of interconversion to fission rates and the degree of anterograde-retrograde asymmetry of particle movement.

4. *Coarsegraining:* From eq. (S3), we can see that the mass profile remains unchanged under the scaling: $L \to \lambda L$, $u \to \lambda^2 u$, $(\gamma - 1/2) \to \lambda^{-1}(\gamma - 1/2)$. This provides a basis for coarsegraining the system, allowing us to reduce the model to a small number of 'effective' lattice sites. However, for the coarsegraining procedure to be meaningful, this effective number should still be large enough that the continuum approximation used in deriving (S3) holds.

S2.2 Structure formation with different types of flux kernels $f[m]$

The above analysis suggests that the flux kernel $f[m^Z]$, which encapsulates how the vesicle fission and conversion rates depend on the mass of the parent aggregate, is a crucial determinant of structure formation. Below we present a detailed comparison of the structures obtained for three different kinds of $f[m^z]$ (represented schematically in figure S3):

I. Mass-independent rates:

$$f[m^Z] = \begin{cases} 0, & \text{if} \quad m^Z = 0 \\ 1, & \text{if} \quad m^Z > 0 \end{cases} \tag{S4a}$$

II. Rate increasing with number m_i^Z of Z particles:

$$f[m^Z] \propto \sqrt{m^Z} \tag{S4b}$$

III. Michelis-Menten type of rates:

$$f[m^Z] = \frac{K_{sat}\sqrt{m^Z}}{\sqrt{m^Z} + \sqrt{m_{sat}}} \tag{S4c}$$

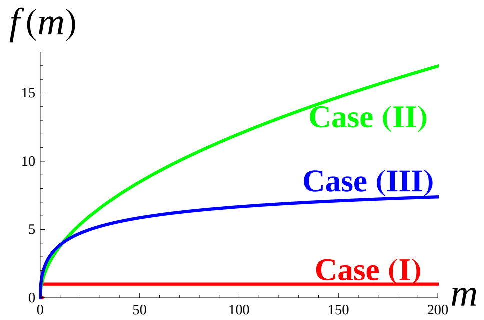

Figure S3: A schematic of the three types of flux kernels $f[m]$ defined in eqs. (S4a)-(S4c).

The above forms of $f[m^Z]$ emerge naturally for processes that are catalyzed by enzymes: If the number of enzyme molecules is much lower than the number of A, B, C particles available for fission or conversion, then the reaction rate is enzyme-limited and roughly independent of $m^A, m^B \dots$ [case (I)]. If the enzyme concentration is in excess of the reactant particles, then the reaction is substrate-limited and the reaction rate is proportional to the number of A, B, C particles available for reaction, leading to $f[m^Z] \propto [m^Z]^\beta$ where $\beta > 0$ [2] [case (II)]. For intermediate enzyme concentrations, we expect Michelis-Menten type of kinetics with the reaction rate increasing as $\sqrt{m^Z}$ for small m^Z and saturating to a constant value at large m^Z [case (III)].

Note that cases (I) and (III) admit runaway growth, as the function $f[m^Z]$ is bounded for large m^Z. Thus, in order to generate finite aggregates with constant mass, we must choose parameters that result in steady state [region in red in fig. S2]. As discussed in sec. S2.1, we can solve for $\langle f[m^Z(x)] \rangle$ in steady state, and approximately derive the mass profiles $\langle m^Z(x) \rangle$ from these solutions, using the following approximations:

$$\langle m^Z \rangle \sim \frac{\langle f[m^Z] \rangle}{1 - \langle f[m^Z] \rangle} \qquad \text{type I } f[m] \tag{S5a}$$

[2] $\sqrt{m^Z}$ rates emerge if we assume that the mass at any location in the 1D model is obtained by integrating over the 2D disc-like structure. A 2D structure with m particles typically has $\sqrt{m}$ molecules at its perimeter. Assuming that only the perimeter molecules are available for reaction, we get reaction rates that are proportional to $\sqrt{m^Z}$

$$\langle m^Z \rangle \sim \langle f[m^Z] \rangle^2 \qquad \text{type II } f[m] \tag{S5b}$$

$$\langle m^Z \rangle \sim \left(\frac{\sqrt{m_{sat}} \langle f[m^Z] \rangle}{K_{sat} - \langle f[m^Z] \rangle} \right)^2 \qquad \text{type III } f[m] \tag{S5c}$$

Equation (S5a) has been derived explicitly for the single-species model without interconversion in [2] and appears to hold in the presence of interconversion as well in the limit $f[m] \to 1$. Equations (S5b)-(S5c) can be derived using the approximation $\langle f[m] \rangle \sim f[\langle m \rangle]$, which is reasonable for $\langle m \rangle \gg 1$. Based on the approximations (S5a)-(S5c) and numerical simulations, we now compare in detail various properties of the structures generated for the three types of $f[m]$. In particular, we consider how the function $f[m]$ governs (i) where compartments form, i.e., the spatial location of the maxima of mass profiles (ii) how localized compartments are, i.e., the width of the maxima (iii) whether compartments are temporally stable, i.e., the fluctuations of mass profiles about the average, and (iv) the robustness of structures to variations in rates, i.e., the relative change in mass profiles due to changes in parameters. Some of these features can also be observed in typical snapshots of structures (fig. S4), where the abundances of A,B,C particles at each location are represented by the heights of the green, red, blue columns at that location.

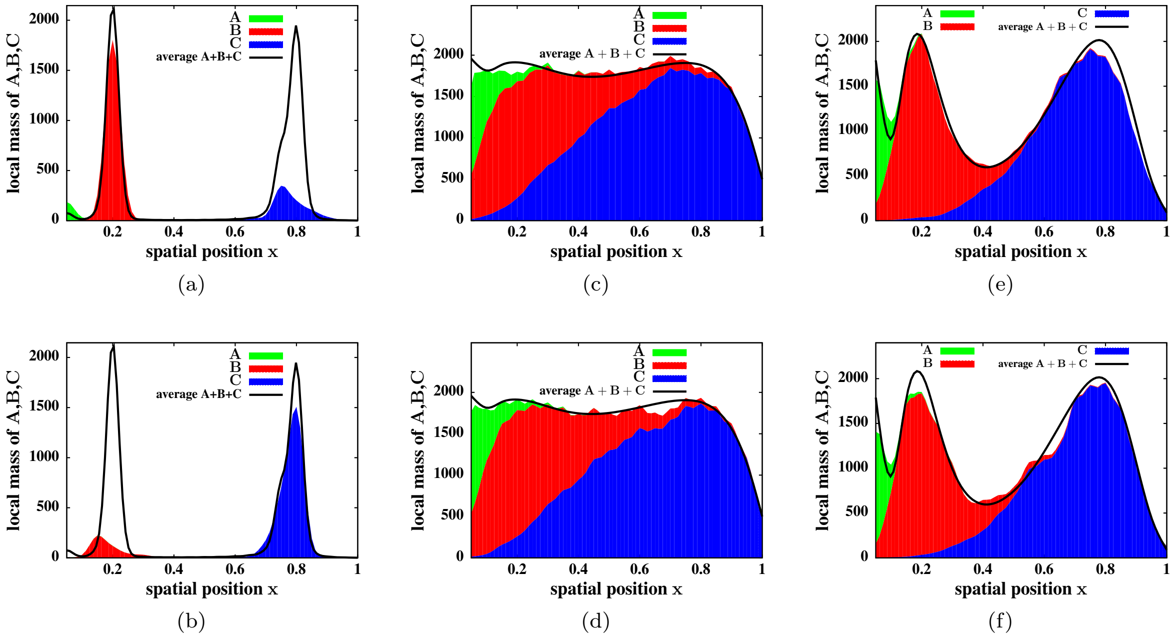

Figure S4: Typical snapshots of the system with pure VT for the three types of $f[m]$ (at two different time instants). (a)-(b): For type I $f[m]$, B, C compartments are well-separated but fluctuate strongly about the average mass profiles (solid lines). (c)-(d): For type II $f[m]$, compartments cannot be resolved into morphologically distinct structures but are very stable in time. (e)-(f): For type III $f[m]$, compartments are both morphologically distinct and reasonably stable in time. Parameters for (a)-(b): a=1, D=0, w_A=0.09368, w_B=0.03346, w_C=0.04175, γ_A=0.5, γ_B=0.6, γ_C=0.66, u=0.0086, v=0.00149, m_{sat}=200, K_{sat}=14.14; (c)-(d): a=1, D=0, w_A=0.03448, w_B=0.01207, w_C= 0.01379, γ_A=0.5, γ_B=0.6, γ_C=0.66, u=0.0031, v=0.00053, m_{sat}=200, K_{sat}=14.14; (e)-(f): a=1, D=0, w_A=0.125, w_B=0.04375, w_C=0.05462, γ_A=0.5, γ_B=0.6, γ_C=0.66, u=0.01125, v=0.00194, m_{sat}=200, K_{sat}=14.14.

Location of maxima: The spatial locations of the maxima of the $\langle m^A \rangle$, $\langle m^B \rangle$, $\langle m^C \rangle, \ldots$ profiles are roughly independent of the form of $f[m]$ and are determined primarily by the rates of the elementary processes in the model. To see this, note that for each of the three forms (I)-(III), the derivative $d\langle m^Z(x) \rangle / dx$ can be zero if and only if the derivative $d\langle f[m^Z(x)] \rangle / dx$ is zero (from eq. (S5)), implying that the maximum

of the $\langle m^Z(x) \rangle$ and $\langle f[m^Z(x)] \rangle$ profiles must coincide. Since the spatial profiles $\langle f[m^Z(x)] \rangle$ depend only on the rates of various processes (see eq. (S3)), and not on the form of $f[m]$, it follows that the corresponding $\langle m^Z(x) \rangle$ profiles and the positions of their maxima must also be independent of the form of $f[m]$.

Width of maxima: In order to obtain non-overlapping compartments, i.e., well-separated peaks in the total mass profile, the width of the maxima must be much smaller than the distance between adjacent maxima. Sharp peaks (with small width) can be obtained only for flux kernels $f[m]$ of types (I) and (III), that saturate at large m. As evident from eqs. (S5a) and (S5c), if the average vesicular fluxes at the peaks locations are close to their saturation value, then the average mass at the peaks is very high. Moreover, in this saturation regime, gentle gradients in the vesicular fluxes, i.e., in $\langle f[m^Z(x)] \rangle$ profiles, can result in very sharp peaks in the $\langle m^Z(x) \rangle$ profiles, thus, leading to sharply localized A-rich, B-rich and C-rich regions (see also sec. S2.1). For $f[m]$ of type (II), there is no such saturation regime; the average mass profiles $\langle m^Z(x) \rangle$ essentially mirror the $\langle f[m^Z(x)] \rangle$ profiles (eq. (S5b)), and have maxima that are wide and difficult to resolve. Average mass profiles for the three types of $f[m]$ are depicted in figs. S4(a)-S4(e) as solid lines, for parameters chosen so as to obtain spatially resolvable peaks (which was not possible in case II). Note that peaks are much sharper in case I [figs. S4(a),S4(b)] than in case III [figs. S4(e),S4(f)].

Stability of mass profiles: To estimate the extent to which mass profiles vary over time, we monitor the rms fluctuations Δm^Z about the average local mass $\langle m^Z \rangle$ of each species at each site. Figure S5(a) shows the relative fluctuations $\Delta m^B / \langle m^B \rangle$ vs. $\langle m^B \rangle$ for the three cases. For mass-independent $f[m]$ (type I), $\Delta m^B / \langle m^B \rangle \sim 1$ for large $\langle m^B \rangle$, so that mass profiles exhibit giant fluctuations about the average (figs. S4(a)-S4(b)). For $f[m]$ of type II and III, where the flux increases with m, the relative fluctuations $\Delta m^B / \langle m^B \rangle$ become smaller as $\langle m^B \rangle$ increases, so that large aggregates are fairly stable in time (see figs. S4(c)-S4(f)). Thus, vesicular fluxes must be sensitive to the mass of the parent aggregate to ensure a a stabilizing negative feedback– when the aggregate becomes too large, the number of particles breaking off from it increases and vice versa, thus tending to restore the aggregate to its average size.

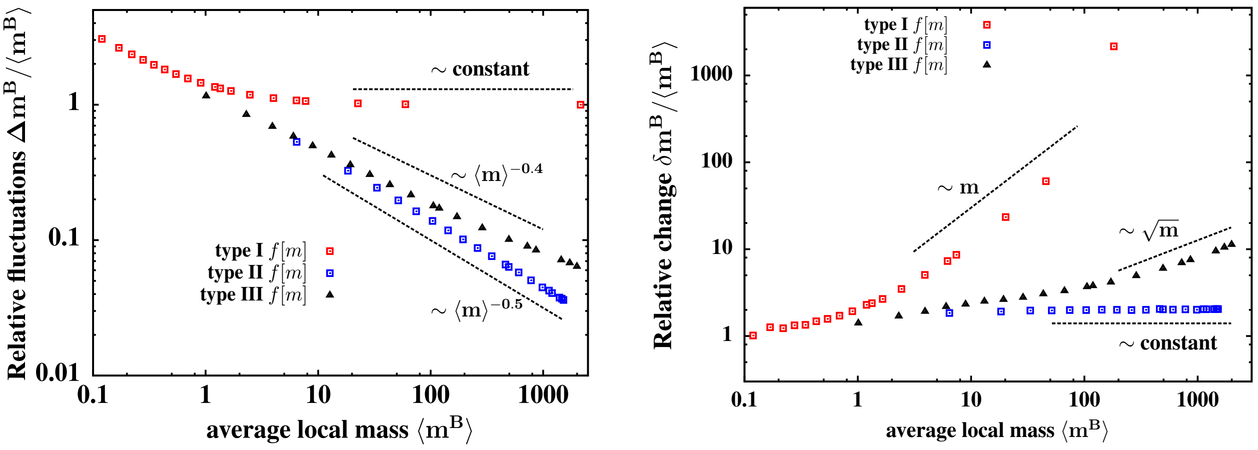

(a) (b)

Figure S5: (a) Log-log plot of relative fluctuations $\Delta m^B / \langle m^B \rangle$ vs. the *local* mass $\langle m^B \rangle$ of B particles. For type I $f[m]$, $\Delta m^B / \langle m^B \rangle \sim 1$, indicating giant fluctuations about the average mass profile. For $f[m]$ of type II and III, $\Delta m^B / \langle m^B \rangle$ decreases with $\langle m^B \rangle$, implying that large aggregates are relatively stable. (b) Log-log plot of the relative change $\delta m^B / \langle m^B \rangle$ at a location with average local mass $\langle m^B \rangle$ of B, due to change in influx. Y-axis is scaled by $\delta a/a$, the increase in influx, where $\delta a/a$=0.005 for $f[m]$ of type I and $\delta a/a$=0.05 for type II and III $f[m]$. The relative change is independent of $\langle m^B \rangle$ for case II, but increases with $\langle m^B \rangle$ for cases I and III. Parameters same as for fig. S4.

Sensitivity of mass profiles to small variations in rates: Changes in model parameters can alter the peak locations of $\langle m^Z(x)\rangle$ profiles as well as peak concentrations. However, in the regime of small interconversion rates ($u, v \ll w_A, w_B$) that we consider here, the locations are relatively insensitive to changes in parameters, and perturbations primarily alter the peak concentrations of some or all $\langle m^Z\rangle$ profiles.

Below we consider how mass profiles respond to a change in influx, by measuring $[\delta m^Z/\langle m^Z\rangle]/[\delta a/a]$ which is the ratio of the relative change $\delta m^Z/\langle m^Z\rangle$ that occurs at a location with average mass $\langle m^Z\rangle$, to the corresponding change $\delta a/a$ in influx. From eqs. (S3) and (S5), it follows that for $\langle m^Z\rangle \gg 1$ and $\delta a/a \to 0$, we expect: $\delta m^Z/\langle m^Z\rangle \propto \langle m^Z\rangle(\delta a/a)$ for $f[m]$ of type I; $\delta m^Z/\langle m^Z\rangle \propto 2(\delta a/a)$ for $f[m]$ of type II; $\delta m^Z/\langle m^Z\rangle \propto 2\left[1+\sqrt{\langle m^Z\rangle/m_{sat}}\right](\delta a/a)$ for $f[m]$ of type III. These expectations are confirmed qualitatively by numerics (fig. S5(b)), with the strongest response to change in influx observed for type (I) $f[m]$, followed by type (III), and the weakest for $f[m]$ of type (II). Furthermore, with type (II) $f[m]$, the relative change in mass is the same in all regions, whereas with $f[m]$ of type (I) and (III), the relative change is more in regions where the local mass is already high. Thus, with type (I) and (III) $f[m]$, the mass profile becomes more/less sharply peaked as influx increases/decreases (see also inset of fig. 5 of main paper). Note that for these types of $f[m]$, a large increase in influx can drive an instability leading to runaway growth– an undesirable feature which is eliminated if there is sub-cisternal movement at a small rate (sec. S3).

This analysis also sheds light on the degree of fine tuning required to generate compartments. Since the mass profiles are most (least) sensitive to small changes in parameters when $f[m]$ is of type I (type II), it follows that the maximum (minimum) degree of fine tuning is required in this case. By the same token, an intermediate degree of fine tuning is required for structure formation with type III $f[m]$.

So far we have considered identical flux control functions for both fission and chemical conversion. However, it is quite plausible that the two processes are governed by different kinetics, for instance if both obey MM kinetics but with different saturation scales m_{sat} and exponents θ in eq. (S2a). Figure S6 depicts structure formation in the pure VT limit, allowing for this possibility, by assuming $\theta=1/2$ for fission (as would be the case if vesicles break off only from the rims of cisternae) and $\theta=1$ for chemical conversion (assuming that Golgi-resident enzymes are distributed throughout the cisterna). While the analytical solutions described above are no longer valid for this case, structure formation and even dynamics is qualitatively similar to the less general scenario (with identical θ and m_{sat}) considered in the main paper.

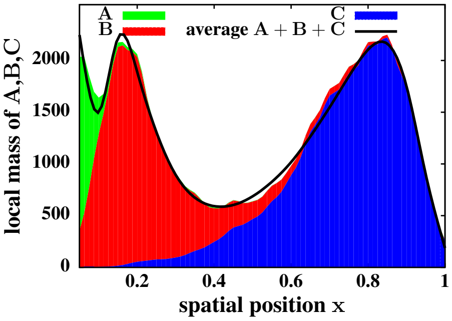

Figure S6: Structure formation with pure VT and MM kinetics for fission and chemical conversion but with $\theta=1/2$ for fission and $\theta=1$ for conversion. Structures are qualitatively similar to the case with the same value of θ for both processes (figs. 4(e), 4(f)). Parameters: $a=1$, $D=0$, $w_A=0.124691$, $w_B=0.049383$, $w_C=0.040741$, $\gamma_A=0.5$, $\gamma_B=0.64$, $\gamma_C=0.724$, $u=0.001062$, $v=0.000136$, $L=20$; parameters governing MM kinetics for fission: $\theta=1/2$, $m^A_{sat}=150$, $K^A_{sat}=12.247$, $m^B_{sat}=200$, $K^B_{sat}=14.14$, $m^C_{sat}=180$, $K^C_{sat}=13.416$; parameters governing MM kinetics for chemical conversion: $\theta=1$, $m^A_{sat}=200$, $K^B_{sat}=14.14$, $m^B_{sat}=150$, $K^B_{sat}=12.247$.

S2.3 Pure VT model with higher number of species

The pure VT model can be generalized to include more species of particles; here we consider a 4-species version with $A\to B\to C\to D$ sequential interconversion. Figure S7(a) shows the average mass profiles for a set of parameters chosen such that the maxima of the B , C and D profiles are sufficiently well separated. The maximum of each mass profile can be made sharp by tuning the maximum of the corresponding $\langle f[m^Z]\rangle$ profile to be close to the saturation value K_{sat}, so that the total mass profile has three distinct peaks

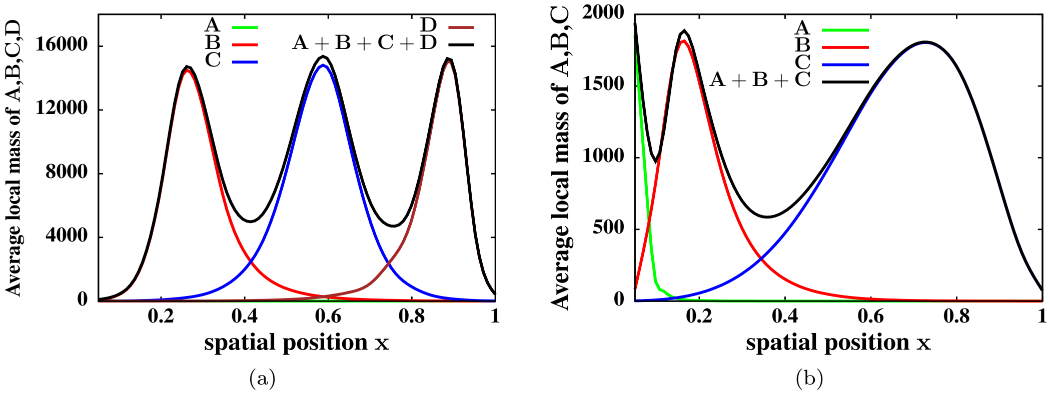

Figure S7: Two variants of the basic model. (a) Four-species model with A,B,C,D particles, sequential $A \to B \to C \to D$ conversion and MM flux kernel. System self-organizes into distinct B, C, D compartments. Parameters: a=1, D=0, w_A=0.3419, w_B=0.12863, w_C=0.00504, w_D=0.00018 γ_A=0.5, γ_B=0.5, γ_C=0.54, γ_D=0.75, u=0.02564, v= 0.000079, r=0.0000071[3], m_{sat}=100, K_{sat}=10. (b) Average mass profiles in the pure VT model with homotypic fusion of A and B particles. Note the similarity with figs. S4(e), S4(f) where there is no homotypic fusion. Parameters: a=1, D=0, w_A=0.15094, w_B=0.03094, w_C=0.03472, γ_A=0.5, γ_B=0.63, γ_C=0.66, u=0.00875, v=0.00146, m_{sat}=200, K_{sat}=14.14, m'_{sat}=100, K'_{sat}=10.

corresponding to morphologically separate B, C and D compartments. The model can be extended in a similar fashion to include more number of species, and thus, obtain a higher number of compartments.

S2.4 Effect of homotypic fusion

We use numerical simulations to study the effect of introducing homotypic fusion in the pure VT model. Homotypic fusion refers to the tendency of an A (or B) vesicle to fuse with a neighboring aggregate with a rate that increases with the number of A (or B) vesicles in the target aggregate. To implement homotypic fusion in our model, we assume that an A particle at site i to hops to its (forward) neighbor j with a rate given by:

$$w_A \gamma_A \left(\frac{K_{sat}\sqrt{m_i^A}}{\sqrt{m_i^A} + \sqrt{m_{sat}}} \right) \left(0.2 + 0.8 \frac{K'_{sat}\sqrt{m_j^A}}{\sqrt{m_j^A} + \sqrt{m'_{sat}}} \right) \tag{S6}$$

The first bracketed term represents enzyme-mediated fission from site i with MM kinetics. The second term represents homotypic fusion of the fissioned A particle with the mass at site j at a rate that increases with the mass of A particles present at the site, but saturates to a constant value beyond a certain mass m'_{sat}. The hopping rates for B particles can be written in a similar fashion.

Figure S7(b) depicts the mass profiles for parameters chosen such that both the fission and fusion terms are operating within the saturation regime. Under this condition, structures generated in the pure VT model with homotypic fusion of A, B (and all others excluding the final product) species, are qualitatively similar to those generated without homotypic fusion [see figs. S4(e), S4(f)]. Thus, in our model, homotypic fusion is not crucial for maintaining compartment identity.

S3 VT-dominated case: Effect of (sub-)cisternal movement

[3] r is the $C \to D$ conversion rate.

We consider a scenario where in addition to vesicular transport, a finite fraction α of an aggregate is allowed to break off and move forward with rate D. This is akin to the Cisternal Progenitor model of [5], and is referred to the VT-dominated limit in the main paper. Breakage and movement of cisternal fragments at a non-zero rate D is sufficient to ensure that there is no runaway growth, even for very large influx rates a. To illustrate this point we analyze a very simple case where the system consists of a single site (L=1), and where there is no interconversion. The average mass on the site evolves as:

$$\frac{\partial \langle m^A(t)\rangle}{\partial t} = a - w_A \langle f[m^A]\rangle - \alpha D \langle m^A\rangle \qquad \text{(S7)}$$

where $f[m^A]$ is of type III [eq. (S4c)].

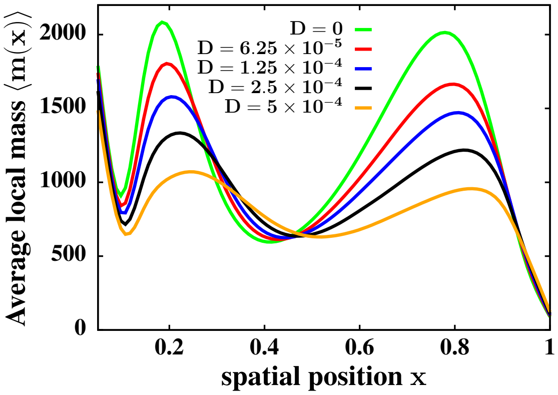

Figure S8: Average mass profiles $\langle m(x)\rangle$ for different values of sub-cisternal movement rate D (cisternal fraction α=0.3 and other parameters same as in figs. S4(e)-(f)). The mass profiles become less peaked with increasing D, making it difficult to resolve adjacent maxima.

For $a/w_A > K_{sat}$ and D=0, the incoming and outgoing flux at the site necessarily fail to balance, as $\langle f[m^A]\rangle$ cannot exceed K_{sat}, leading to runaway growth of mass. Now consider the case with non-zero D. As $\langle m^A\rangle$ increases, the average outgoing flux (which includes the term $\alpha D\langle m^A\rangle$) also increases, until it balances the incoming flux a. At this point, $d\langle m^A\rangle/dt$ must become zero, ensuring that $\langle m^A\rangle$ is constant at long times. Thus, as long as the outgoing flux has a component that *keeps increasing with the mass* m at the site, there can be no runaway growth.

In order to preserve the well-separated compartments that emerge in the pure VT model, the rate D must be smaller than the single particle fission rates. As D increases, the peaks in the mass profile become shallower, until adjacent peaks can no longer be resolved (fig. S8). Thus, we consider VT-dominated scenarios where cisternal movement acts as a secondary track to vesicular transport. This eliminates runaway growth while maintaining sharp peaks in the mass profile (see also figs. 2(c) and 2(c') of the main paper).

S4 Typical steady state configurations in aggregate representation

Steady state configurations of mass for various transport models (see fig. 2 of the main paper) can also be shown in an alternative 'aggregate representation' where the local mass of A, B, C at spatial position x is represented by the heights of the green, red, blue columns at x. This representation is particularly useful in tracking changes in mass due to perturbations, for instance in movies S9-S12, or for detecting fluctuations of the mass profiles about the time-averaged concentrations (see solid black lines in fig. S9, also fig. S4).

S5 Dynamical measurements

We consider below two kinds of dynamical measurement which address: (i) how the number of tagged particles decays in time after the full system is tagged at t=0, and (ii) how the compositional entropy changes during reassembly of the system from an unpolarized state.

S5.1 Dynamics of (fluorescently) tagged particles

We perform measurements inspired by iFRAP (inverse Fluorescence Recovery After Photobleaching) experiments in which the cargo pool in the entire Golgi region is fluorescently tagged and highlighted and then the subsequent decay of fluorescence monitored to infer the exit kinetics of the cargo molecules [3]. The observation of exponential decay of fluorescence in these experiments has been used to argue against

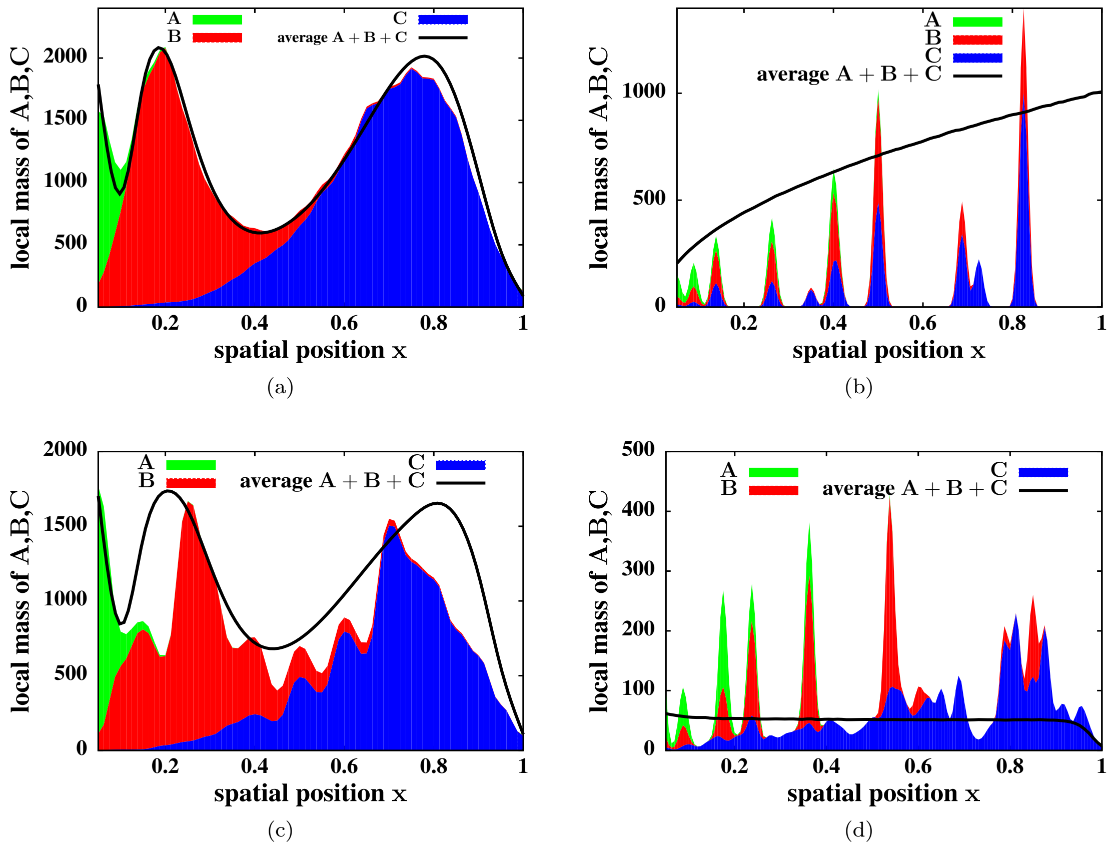

Figure S9: Typical steady state configurations in the aggregate representation for (a) pure VT (b) pure CP (c) VT-dominated transport with rare sub-cisternal movement, akin to the Cisternal Progenitor model and (d) CP-dominated transport with fission of single C particles. Solid black lines represent time-averaged profiles of the total mass. All parameters same as in fig. 2 of main paper.

the cisternal progression hypothesis [3]. To further explicate these findings, we implement a similar protocol in our model, by tagging all the particles present in the system at an arbitrary time instant $t=0$ in steady state, and monitoring how the proportion of tagged particles decays in time. Figure S10(a) shows $M_{tag}(t)/M_{tag}(0)$ (averaged over 100 ensembles) for each of the four cases - pure VT, pure CP, VT-dominated and CP-dominated. A clear linear decay in tagged particle concentration occurs only for the pure CP case. Exponential decay is observed for both the pure VT case and the VT-dominated case where cisternal movement provides a secondary track for traffic. Further, even in the CP-dominated case, where the bulk of the transport is through cisternal progression, there is a significant deviation from linear decay. This suggests that the observation of exponential decay in fluorescence experiments is consistent with cisternal progression as long as there is concomitant vesicular movement. A similar conclusion is reported in [4] where the iFRAP experiments in [3] were quantitatively analyzed to show that the exponential decay can be explained by a number of alternative scenarios involving vesicular efflux in addition to cisternal movement.

S5.2 Dynamics of reconstitution after disassembly

We consider below the dynamics of re-assembly of the system in the pure VT and pure CP scenarios, and in the process also distinguish reassembly from de novo formation. In our terminology, reassembly refers to the reconstitution of the system from an initial state in which the molecules and markers that comprise

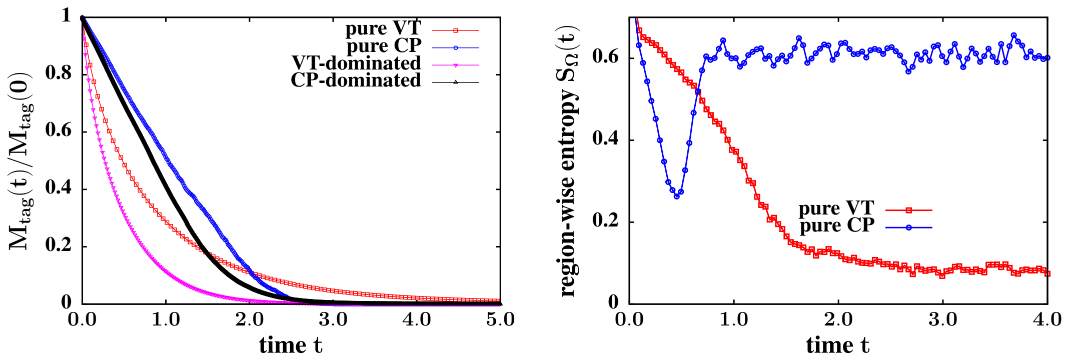

(a) (b)

Figure S10: (a) Fraction of tagged particles $M_{tag}(t)/M_{tag}(0)$ remaining in the system at time t vs. t (obtained by averaging over 100 ensembles). Scale of X axis is $1 \equiv 10^5 t.u.$ for pure VT and VT-dominated cases, $1 \equiv 10^4 t.u.$ for the pure CP case and $1 \equiv 5 \times 10^3 t.u.$ for the CP-dominated case. $M_{tag}(t)/M_{tag}(0)$ decays linearly in the pure CP case but deviates from linear decay in CP-dominated case. Exponential decay for pure VT and VT-dominated cases. (b) Compositional entropy $S_\Omega(t)$ vs. t for the region Ω:$0.5 < x < 0.75$ during reassembly from an unpolarized state (averaged over 100 ensembles). Scale of X axis is $1 \equiv 10^4 t.u.$. $S_\Omega(t)$ decreases during reassembly for pure VT case but changes non-monotonically in the pure CP case. All parameters same as in figs. 2 and 3 of main paper.

the cisternae are all present in the Golgi region but distributed in a uniform, unpolarized manner. De novo formation, on the other hand, refers to the re-formation of structures starting from an initially 'empty' state, i.e. with molecules resorbed into the ER and/or dispersed far into the cytoplasm.

To study re-assembly in Monte Carlo simulations, an initial state is constructed by redistributing all the A,B,C particles (that constitute the steady state structures for a specific set of rates) uniformly across the system. We then switch on all transport and interconversion processes at the same rates and monitor how the number of A,B,C particles in different regions evolves in time (see also movies (S7) and (S8)). As in the case of de novo formation, this information can be used to compute the region-wise compositional entropy $S_\Omega(t)$ (see main paper for a formal definition). As the structures re-assemble, $S_\Omega(t)$ decreases with time in the pure VT case and changes non-monotonically in the pure CP cases for the region $0.5 < x \leq 0.75$ (fig. S10(b)). In general, $S_\Omega(t)$ can show complex non-monotonic behaviour in both limits depending on the region of interest. This is in contrast with de novo formation where the dynamics of the region-wise entropy provides a clear way of discriminating between the two mechanisms, increasing with t during formation in the pure CP case, and decreasing with t in the pure VT case (see fig. 4(f) of the main paper).

The contrast in the dynamics of $S_\Omega(t)$ during de novo formation and reassembly in the pure CP limit highlights the sensitivity of dynamical measurements to the initial state of the system. The difference between the two dynamics can be rationalized as follows. During de novo formation, early compartments mature into medial and then trans compartments, thus creating mixed compartments, and increasing the compositional entropy. Eventually as this process goes on, the creation of mixed compartments due to maturation of early compartments is balanced by the loss of mixed compartments due to maturation into late compartments, so that S_Ω reaches a time-independent value. However, in the early stages of de novo formation, when this balance has not yet been achieved, $S_\Omega(t)$ increases with time. In the case of reassembly, on the other hand, early as well as late components are already present in the system in a completely random, unpolarized manner. Reassembly thus essentially involves the re-emergence of polarity in the system, and is thus qualitatively different from de novo formation.

S6 Captions for Supplementary Movies (S1)-(S14)

Steady state fluctuations [(S1)-(S4)]
Movies (S1)-(S4) follow the self-organized structures in each of the 4 scenarios (pure VT (S1), VT-dominated (S2), pure CP (S3), CP-dominated (S4)) over a period of time, and provide a window into the dynamics and fluctuations of these structures in steady state. The structures are represented by separate intensity plots for each of the three species $A, B, C \ldots$, with the intensity of green at any spatial position x being proportional to the abundance of A particles at x and so on.
(S1): In the pure VT limit, A, B, C particles are stably localized in different region of the system. The abundance of particles in any region shows only minor variation over time.
(S2): In the VT-dominated limit, there is greater variation over time, with small chunks breaking off from one structure and fusing with the next (Note the movement of light red plus light blue bars between the B-rich and C-rich compartments).
(S3): In the pure CP limit, there is a high turnover of cisternae– any macroscopic region of the system has large mobile cisternae entering and exiting, leading to strong fluctuations in molecular abundances over time. It is also possible to visualize the maturation process by following an individual cisterna over time– a typical cisterna changes color from primarily green near the cis end to green-red to red-blue as it moves, becoming primarily blue near the trans end.
(S4): The CP-dominated limit is qualitatively similar to the pure CP limit, except near the trans end where cisternae disintegrate into smaller fragments before leaving the system (Contrast the light blue bars near the trans end in the CP-dominated limit with the intense blue bars in the pure CP limit).

De novo formation [(S5)-(S6)]
Movies (S5)-(S6) show how structures form *de novo* in the pure VT limit (S5) and pure CP limit (S6), starting from an initial condition in which the system is completely ‘empty’.
(S5): In the pure VT limit, compartment formation involves a long period of accumulation of A, B and C particles in separate regions of the system, with no mixed compartments forming. A, B, and C compartments regenerate over different timescales, with the cis (A) compartment taking the shortest and the trans (C) compartment taking the longest time to form.
(S6): In the pure CP limit, the system regenerates over a time scale that is the same as the time required for cisternae to traverse the system (see movie S3). Regeneration is characterised by the formation and movement of mixed (two-color) compartments.

Reassembly dynamics [(S7)-(S8)]
Movies (S7)-(S8) show how cisternae reassemble in the pure VT limit (S7) and pure CP limit (S8), starting from an unpolarized state in which all structures are dissipated and their contents uniformly dispersed across the system.
(S7): In the pure VT limit, compartments reassemble through the localization and aggregation of particles of different species (colors) in different regions.
(S8): In the pure CP limit, compartment formation involves two stages– an initial ‘fast’ phase in which all types of particles come together into small mixed aggregates, and a subsequent longer phase in which chemical polarity re-emerges as the A-rich structures formed from freshly injected particles traverse the system, maturing into B-rich and then C-rich compartments in different stages of their progression.

Response to change in influx [(S9)-(S12)]
Movies (S9)-(S10) show how the mass profiles change in response to a small drop (S9) or rise (S10) in influx in the pure VT limit. (S11)-(S12) show the corresponding response to a small drop (S11) or rise (S12) in the pure CP limit. To depict changes in mass accurately, we use an alternative representation in which the number of particles of type A, B, C at a spatial position is proportional to the height of the green, red or blue column respectively at that position.
(S9)-(S10): A small change in influx (10% drop in (S9) and 6.25% rise in (S10)) causes the mass profile to

shift significantly from its unperturbed state (solid black line). The proportionate change is higher near the peaks of the mass profiles than at the valleys. Different compartments respond over appreciably different time scales, with the cis cisternae attaining their new size much earlier than the trans cisternae. The systematic change in cisternal size is easily distinguishable from steady state fluctuations.
(S11)-(S12): A 20% drop (S11) or rise (S12) in influx causes a modest change in cisternal size. Dashed and solid black lines represent the typical cisternal size (obtained by averaging over many ensembles) in the unperturbed state and after a 20% drop/rise in influx respectively. Note how the cisternae in the movies can be significantly larger or smaller than this average size making it difficult to distinguish the systematic change in size due to altered influx from usual stochastic fluctuations.

Response to exit block [(S13)-(S14)]
Movies (S13)-(S14) show how cisternae respond to a blockade of the exit site at the trans end in the pure VT (S13) and pure CP (S14) limits. In both limits, exit blocks induce a piling up of particles at the exit site, with little or no change in the rest of the system. In our model, this pile-up continues indefinitely in time, thus pointing towards the need for an additional mechanism that stabilizes the enlarged trans cisterna in the presence of an exit block.

References

[1] H. Sachdeva, M. Barma and M. Rao, Phys. Rev. E **84**, 031106 (2011).

[2] E. Levine, D. Mukamel and G. M. Schütz, J. Stat. Phys. **120**, 759 (2005).

[3] G.H. Patterson, K. Hirschberg, R.S. Polishchuk, D. Gerlich, R.D. Phair and J. Lippincott-Schwartz, Cell **133**, 1055 (2008).

[4] S. Dmitrieff, M. Rao and P. Sens, Proc. Natl. Acad. Sci. (USA), **110**, 15692-15697 (2013).

[5] S.R. Pfeffer, Proc. Nat. Acad. Sci. (USA), **107**:19614-19618 (2010).
